# Sex-dependent differences in hematopoietic stem cell aging and leukemogenic potential

**DOI:** 10.1038/s41388-024-03197-9

**Published:** 2024-11-01

**Authors:** Chunxiao Zhang, Taisen Hao, Alessia Bortoluzzi, Min-Hsuan Chen, Xiwei Wu, Jinhui Wang, Richard Ermel, Young Kim, Shiuan Chen, WenYong Chen

**Affiliations:** 1 Department of Cancer Biology and Molecular Medicine, Beckman Research Institute, City of Hope, Duarte, CA 91010 USA; 2 Integrative Genomics Core, Department of Molecular and Cellular Biology, Beckman Research Institute, City of Hope, Duarte, CA 91010 USA; 3 Center for Comparative Medicine, Beckman Research Institute, City of Hope, Duarte, CA 91010 USA; 4 Department of Pathology, City of Hope National Medical Center, Duarte, CA 91010 USA; 5 Present Address: Amgen, Thousand Oaks, CA USA; 6 Present Address: Bristol Myers Squibb, Seattle, WA USA

**Keywords:** Ageing, Acute myeloid leukaemia, Cancer stem cells, Cancer models

## Abstract

Sex influences many biological outcomes, but how sex affects hematopoietic stem cell (HSC) aging and hematological disorders is poorly understood. The widespread use of young animal models to study age-related diseases further complicates these matters. Using aged and long-lived BALB/c mouse models, we discovered that aging mice exhibit sex-dependent disparities, mirroring aging humans, in developing myeloid skewing, anemia, and leukemia. These disparities are underlined by sex-differentiated HSC aging characteristics across the population, single-cell, and molecular levels. The HSC population expanded significantly with aging and longevity in males, but this occurred to a much lesser degree in aging females that instead expanded committed progenitors. Aging male HSCs are more susceptible to BCR-ABL1 transformation with faster development of chronic myeloid leukemia (CML) than female HSCs. Additionally, the loss of the aging regulator Sirt1 inhibited CML development in aging male but not female mice. Our results showed for the first time that sex-differentiated HSC aging impacts hematopoiesis, leukemogenesis, and certain gene functions. This discovery provides insights into understanding age-dependent hematological diseases and sex-targeted strategies for the treatment and prevention of certain blood disorders and cancer.

## Introduction

Aging affects men and women differently. Women live longer but paradoxically face greater frailty and health issues late in life [1]. The blood system, common to both sexes, shows sex disparities in autoimmune diseases prevalent in women [2]. Hematological aging in humans is characterized by skewed differentiation toward the production of more myeloid cells and fewer lymphocytes, particularly B cells [3, 4]. Estrogen suppresses B cell production [5], yet post-menopausal women still produce fewer B cells. Anemia impacts about 17% of seniors >65 years globally, affecting millions especially those hospitalized [6, 7]. Anemia contributes to frailty, cognitive impairment, and cardiovascular disease in the seniors [6]. Women have a greater incidence of moderate to severe anemia with worse clinical presentations than men [8–10]. Conversely, myeloid leukemias, both acute (AML) and chronic (CML), occur more often in aging men [11, 12]. Female CML patients have more favorable prognoses and molecular responses to tyrosine kinase inhibitors (TKIs) [13, 14]. However, the mechanisms underlying these sex-based differences in these blood disorders are poorly understood.

Aging of HSCs is believed to be a key factor driving hematological aging [15]. Aging HSCs exhibit increased cell cycling, resulting in an expansion of HSCs and myeloid-biased differentiation [16, 17]. Aging HSCs undergo transcriptome changes, with clonal expansion of myeloid-biased cells (Refs [18–21] and additional refs in the meta-analysis by Flohr Svendsen et al. [22]). Sex is a historically overlooked variable in biomedical research [23] because of the fear that female cyclic hormone fluctuations may introduce additional variation [24]. Likewise, sexes are often not disclosed or segregated in mouse studies of HSC aging. Despite recent reports showing that estrogen and pregnancy affect HSC self-renewal in young female mice [25, 26], sex’s impact on HSC aging is unclear. This gap is compounded by the prevalent use of young lab animals for age-related disease studies. The impact of model organisms’ age on disease risk, intervention and therapeutic outcome has been under-researched, especially when sex is factored in.

The current knowledge of HSCs and their functions is mostly gained from C57BL/6 mice [15]. However, multiple lines of evidence suggest that BALB/c mice are a good model for hematological aging research. Their sex-based lifespan differences mirror human patterns, with females living longer than males [27]. This provides an advantage over C57BL/6 mice, in which males have a longer lifespan [27]. BALB/c mice possess a hypomorphic p16^INK4a^ allele [28, 29], simulating human p16^INK4a^ inactivation during aging. Female BALB/c mice exhibit higher CD4^+^ and CD8^+^ T cell counts than males [30, 31], paralleling human immune profiles [32, 33]. From a practical point, BALB/c mice have shorter lifespans than C57BL/6 mice [27], facilitating more manageable aging studies. Additionally, BALB/c mouse models of CML have been well established including a recent aging mouse model of CML in 75% of lifespan that more closely mimics human CML in the elderly with increased anemia incidence [34].

Using aging BALB/c mouse models, here we demonstrated sex-dependent differences in the development of skewed myeloid differentiation, hemolytic anemia, and leukemia in aging mice in a way similar to that of aging humans. These were underlined by the sex-dependent differences of HSC aging in which aging males had a greater expansion of HSCs than did aging females. Single-cell RNA-seq (scRNA-seq) revealed several common HSC aging pathways across mouse strains and sexes, but IFNα response, sex hormone signaling and autoimmune pathways were discerning for male and female aging HSCs. We showed that old male BALB/c mice developed CML significantly faster than females did, and Sirt1 deletion inhibited CML in aging males but not females. Our results underscore sex as a crucial factor influencing HSC aging, leukemogenesis, and Sirt1 functions in aging HSC transformation.

## Results

### Sex-dependent differences in hematological aging

**Fig. 1:**
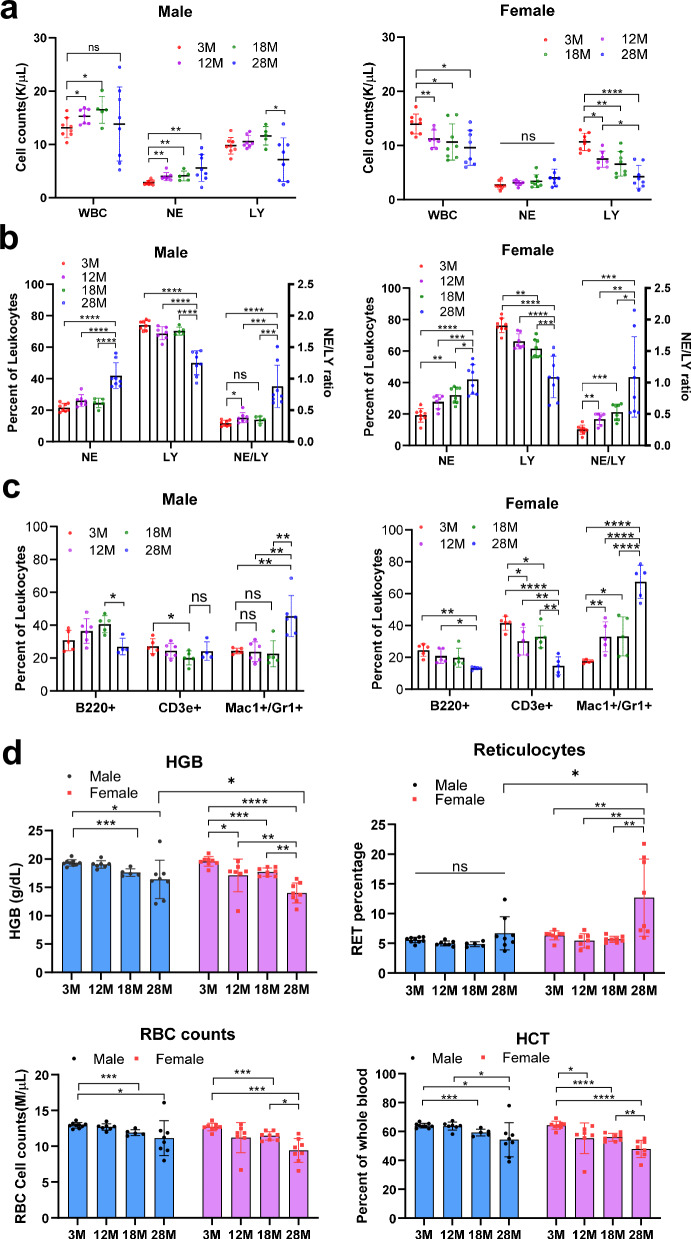
Sex-dependent differences in hematological changes in aging and long-lived BALB/c mice. **a** Differential blood leukocyte counts of males and females aged 3, 12, 18, and 28 months (M) were analyzed via an Idexx ProCyte Dx hematology analyzer. WBC, white blood cell; NE, neutrophil; LY, lymphocyte. **b** The percentages of NE and LY cell counts and their ratios. **c** Peripheral blood leukocytes were analyzed by flow cytometry. **d** Erythrocyte analysis of hemoglobin (HGB) levels, red blood cell (RBC) counts, reticulocyte (RET) percentage, and hematocrit (HCT) in males vs females at different ages by Idexx ProCyte Dx. *p < 0.05; **p < 0.01; ***p < 0.001; ****p < 0.0001; ns, not significant. The error bars represent one standard deviation.

We established an aging colony of BALB/c nonbreeder mice. Grossly normal mice without obvious illness signs were chosen for the study as described [34]. We examined hematological changes in males and females from young (3 months), aged/aging (18 months, 75% median lifespan), to long-lived (28 months, >110% median lifespan) mice. We found that aging in males was associated with increased white blood cell (WBC) counts, which were associated with increased neutrophil (NE) counts until 28 months, when some males lost lymphocyte (LY) counts substantially. In contrast, females exhibited reduced WBCs with age due to progressive lymphocyte loss. (Fig. 1a). Percentagewise, females had more persistent LY decrease and NE increase with age (Fig. 1b). Flow cytometry confirmed greater T (CD3e^+^) and B (B220^+^) cell decline in females, with male B cells dropping significantly only in late life (Fig. 1c). Skewed myeloid differentiation was observed in both sexes by 28 months but occurred earlier in females. Our findings are reminiscent of early reports that men’s WBCs increase with more myeloid cells toward aging, while women’s decrease with significant lymphocyte loss [35, 36].

BALB/c mice developed mild anemia at 18 months, worsening by 28 months, as indicated by reduced hemoglobin levels, red blood cell counts and hematocrit levels (Fig. 1d). Female mice at 28 months exhibited more severe anemia, with lower hemoglobin and higher reticulocyte percentage than males (Fig. 1d). Anemia with reticulocytosis suggests hemolytic anemia from autoimmune destruction of red blood cells [6]. The sex differences in the BALB/c anemia phenotypes mirror human trends, where moderate to severe anemia is more prevalent in females, particularly older women with pronounced clinical symptoms [8–10].

When the above data were re-plotted by comparing male and female blood cells at each time point, overall differences for blood cell counts were less pronounced between the two sexes, but declining B cells, anemia and increasing monocytes were more consistently observed in aged females (Supplementary Fig. 1). Yet this comparison obscured certain age-related changes unique to each sex and it will be further discussed below in molecular analysis.

### Sex-dependent differences in hematological aging were hematological cell autonomous

**Fig. 2:**
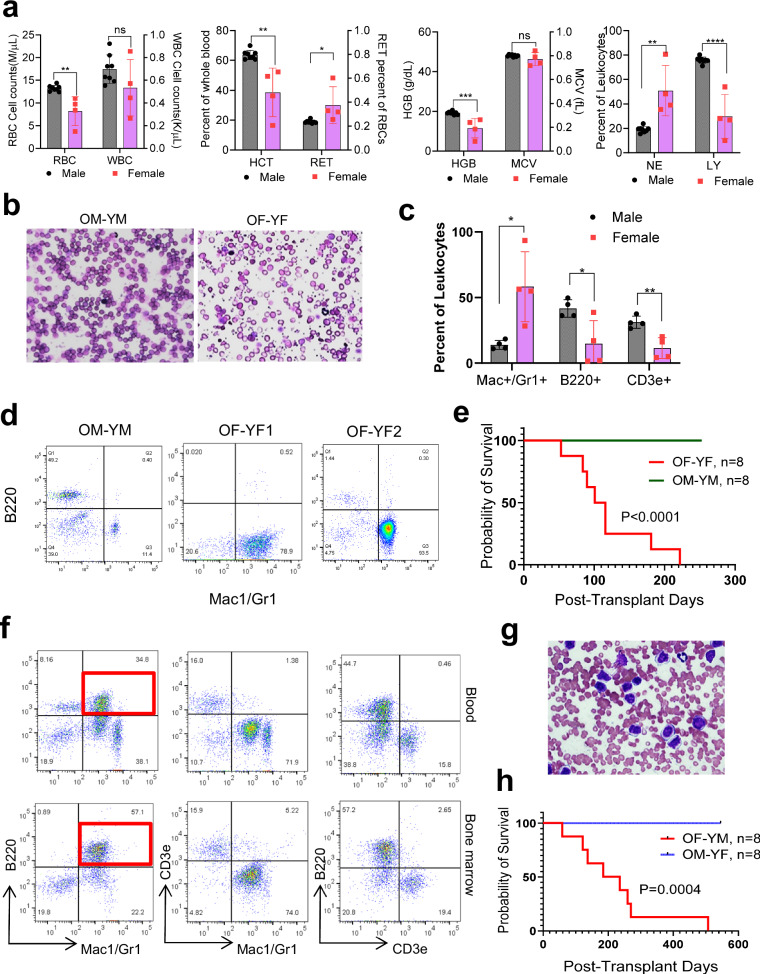
Sex-dependent differences in hematology and survival in primary BMT recipients with donor cells from long-lived mice. **a**–**e** Same-sex BMTs of 28-month-old donor cells to 3-month-old recipients. Peripheral blood cell counts (**a**), blood smear (**b**), flow cytometry analysis of lineage cells (**c**), flow cytometry profiles of skewed myeloid differentiation (OF-YF1) and AML (OF-YF2) compared to a male recipient (**d**), and Kaplan‒Meier survival curves (**e**). **f**–**h** Cross-sex BMTs of 25-month-old female and 28-month-old male donor cells to 3-month-old recipients. Flow cytometry analysis of blood and bone marrow cells (**f**) and blood smears (**g**) from a leukemic OF-YM mouse and survival curves for cross-sex BMTs (h). *p < 0.05; **p < 0.01; ***p < 0.001; ****p < 0.0001; ns, not significant. The error bars represent one standard deviation.

We examined whether sex-dependent differences in hematological aging were intrinsic to hematological cells. We first performed total bone marrow transplantation (BMT) of 28-month-old mouse donors to 3-month-old recipients of the same sex. Healthy long-lived mouse bone marrow (BM) samples from both sexes with comparable profiles (Supplementary Fig. 2a) were used. As shown in Fig. 2a–c, old female-to-young female (OF-YF) BMT recipients exhibited greater myeloid skewing and anemia than did old male-to-young male (OM-YM) BMT recipients, similar to that in 28-month-old mice. All female recipients succumbed to anemia or acute myeloid leukemia (AML) within 8 months post-transplant; male recipients, however, remained healthy (Fig. 2d, e and Supplementary Fig. 2b, c).

**Fig. 3:**
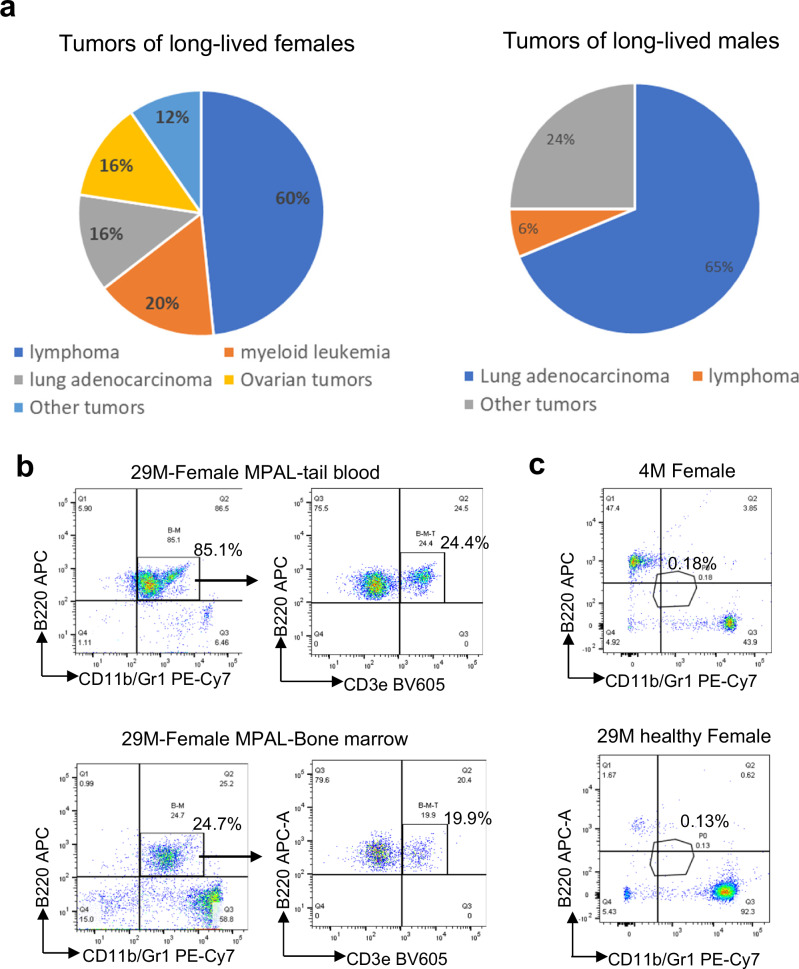
Sex-dependent differences in the tumor spectrum between long-lived male and long-lived female mice. **a** Complete histological analysis was performed for diseased long-lived mice (25 females and 17 males) with an average age of 28.2 months. The percentage indicates the incidence of tumor categories among the mice analyzed. Some mice had more than one tumor type. In females, myeloid leukemia included AML and B/myeloid MPAL; ovarian tumors included ovarian carcinoma and hemangiosarcoma; and other tumors included adenocarcinoma and soft tissue sarcoma in other organs. In males, other tumors included renal cell carcinoma, hepatoma, hemangiosarcoma and adenocarcinoma. **b**, **c** Flow cytometry analysis of a long-lived female BALB/c mouse bearing B/M MPAL that was partially CD3e positive (**b**) compared to the tail blood of 4-month-old and healthy 29-month-old females, with the latter displaying strong myeloid skewing (**c**).

Because of the difficulty in confirming donor cells in the same-sex BMT, we carried out cross-sex BMT with long-lived mouse donor cells (Supplementary Fig. 3a). Both old male-to-young female (OM-YF) and old female-to-young male (OF-YM) BMTs were confirmed by SRY genotyping of recipient WBCs (Supplementary Fig. 3b). Interestingly, in OF-YM BMTs, female Xist gene expression was significantly suppressed in all recipients (Supplementary Fig. 3c). We found that OF-YM BMT mice developed predominantly B/myeloid mixed phenotype acute leukemia (B/M MPAL), a leukemia of hematopoietic stem/progenitor cell (HSPC) origin [37], which was defined morphologically and phenotypically by ≥20% blasts in peripheral blood or bone marrow that were positive biphenotypically for both B (B220^+^) and myeloid lineage (Mac1/Gr1^+^) markers (Fig. 2f, g and Supplementary Fig. 3d), as described previously [38]. As a result, OF-YM BMT mice died quickly, while OM-YF BMT mice were healthy (Fig. 2h). B/M MPAL developed in OF-YM BMT mice with a cumulative BM age similar to what we showed previously with aging male donor cells that require serial BMT to produce B/M MPAL in young female recipients [38]. The high incidences of B/M MPAL occurred in 4 out of 5 independent BMT experiments with donor cells from 25- to 29-month-old females in 36 young recipient mice. Our data suggest that sex-dependent disparities in hematological aging and leukemogenesis are intrinsic to hematological cells and that aging female HSPCs are inherently more prone to developing differentiation defects and MPAL or AML than males. In line with this, long-lived female mice developed predominantly hematological malignancies, including splenic lymphoma, B/M MPAL and AML, while long-lived males developed predominantly lung adenocarcinoma (Fig. 3). The development of spontaneous B/M MPAL and AML in long-lived females suggested that leukemia-initiating cells may preexist in old females before BMT. Intriguingly, no significant changes in BM cellularity or excessive adipogenesis were observed in long-lived BALB/c males or females, although dilated sinusoids of the BM were observed (Supplementary Fig. 4).

### Sex-differentiated HSC aging

**Fig. 4:**
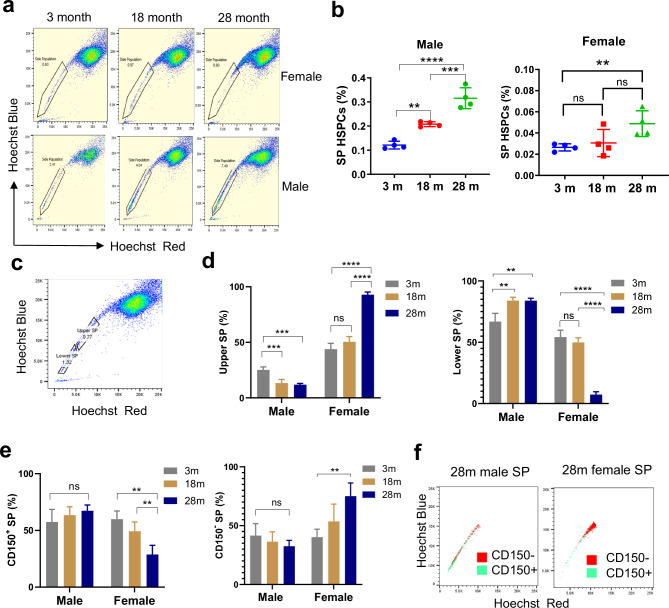
Sex-dependent differences in hematopoietic stem/progenitor cells from aging and long-lived BALB/c mice. **a** Representative flow cytometry plots of bone marrow SPs from male and female BALB/c mice at different ages. **b** Frequencies of SP HSPCs in males vs females in 3-, 18- and 28-month (m)-old mice. Notice the scale difference between males and females. **c**, **d** Gating for the upper and lower SP fractions (**c**) and the percentage of upper and lower SP fractions within the total SP pool (**d**). **e** Frequencies of CD150^+^ and CD150^-^ SP HSCs in males vs females in 3-, 18- and 28-month-old mice. **f** Distribution of CD150^+^ and CD150^-^ cells within the SP pool in 28-month-old mice. *p < 0.05; **p < 0.01; ***p < 0.001; ****p < 0.0001; ns, not significant. The error bars represent one standard deviation.

We next examined changes in BALB/c HSCs during aging. The widely used HSC cell markers Sca-1 and CD150 do not enrich long term (LT)-HSCs effectively in BALB/c mice [39, 40]. HSCs of BALB/c mice reside exclusively in the lineage-negative side population (shorted as SP) and both CD150^+^ and CD150^-^ SP fractions contain LT-HSCs [40]. SP has been shown to effectively track HSCs during aging [18, 41]. Toward aging, BALB/c mice showed sex-specific changes in SP cells (Fig. 4a). Males maintained greater SP percentages throughout life (Fig. 4b), with lower SP fractions increasing from 18 through 28 months (Fig. 4c, d). Females’ SP remained stable till 18 months, then upper SP fractions surged by 28 months (Fig. 4c, d). This created a polarized SP pattern in mice at 28 months with males skewing lower and females higher. Furthermore, CD150^+^ SP cells moderately increased in aging males, while dramatically decreasing in females by 28 months (Fig. 4e). Conversely, CD150^-^ SP cells spiked in long-lived females (Fig. 4e). CD150^+^ SP cells were predominantly localized toward the lower SP in long-lived males, with CD150^-^ SP evenly distributed. Long-lived females showed even CD150^+^ SP distribution, but CD150^-^ SP cells concentrated in the upper fractions (Fig. 4f). The changes in aging BALB/c male HSCs mirror previous findings of expanding lower SP cells during aging, which are enriched in CD150^+^ HSCs with myeloid-biased lineage potential [18]. Our finding of highly CD150^-^ enriched upper SP in long-lived females is intriguing. While CD150^-^ SP also contains long-term HSCs [40, 41], its biological significance remains unknown. Our data suggest that CD150^-^ SP cells may maintain hematological functions in long-lived females.

### Sex-dependent distinctions in the molecular signatures of HSC aging

**Fig. 5:**
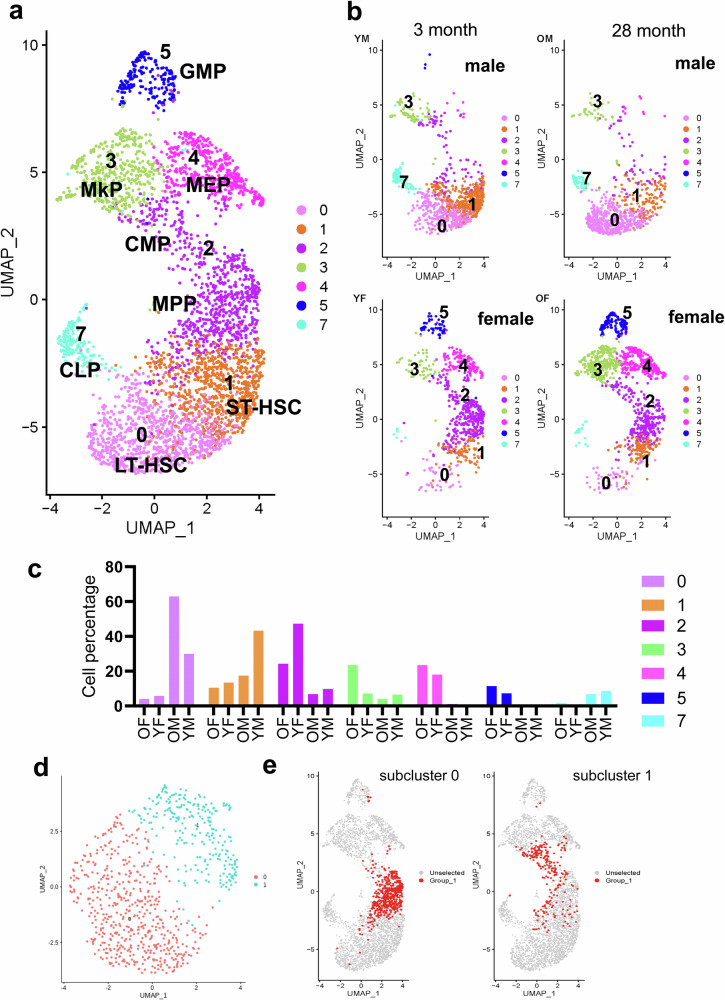
Sex-dependent differences in aging mouse HSCs according to scRNA-seq analysis. **a** Main clusters in the aggregate of all samples (3- and 28-month-old males and females). Overall, 8 clusters were assigned to the aggregates of all the samples, with the majority of cells forming continuous clusters, except for cluster 6, which was a minor cluster distal to the main SP population and likely more differentiated cell contaminants. Cluster 6 was removed from the view but can be found in Supplementary Fig. 5. **b**, **c** Split view of the main SP clusters in separate samples according to sex and age (**b**) and the percentage of each cluster (**c**). YM, young male; OM, old male; YF, young female; OF, old female. **d**, **e** Cluster 2 was reclustered into two subclusters (**d**) that exhibited different distributions in the aggregated UMAP (**e**). Clusters were assigned to HSPCs according to key gene expression features as follows: cluster 0 for LT-HSCs that were negative for the surface markers CD48 and Flt3; cluster 1 for ST-HSCs that closely shared many signature genes with cluster 0 but had an increase in Flt3 and CD34 expression; cluster 2 had stronger activation of Flt3 and CD34; the lower part of cluster 2 (mostly subcluster 0) was assigned to MPP and the upper part (mostly subcluster 1) was assigned to CMP; clusters 3 and 4 were assigned to MkP and MEP, respectively, as both enriched for platelet genes, including Itga2b and Gp1bb; however, cluster 4 was less cycling and had lower Flt3; cluster 5 for GMP that was enriched for the marker genes Mpo, Ctsg, Elane, Prtn3 and Ms4a3; and cluster 7 for common lymphoid progenitors (CLPs) that had a UMAP position close to clusters 0 and 1 but carried its signature genes regulating B and T lymphoid cell development, including Erg1, Fos, Nr4a1, Nr4a2 and Nfkbia. In contrast, the MPP and CMP cells were less well defined by gene signatures.

To better understand the mechanisms of HSC aging, we performed scRNA-seq of SP cells. SP cells were purified from healthy 3- and 28-month-old male or female mice, with 2 to 4 mice/group pooled to reduce individual mouse variations and increase cell yields. An average of 1000 cells per sample with satisfactory libraries were sequenced, and approximately 3500 genes were detected per cell (Supplementary Fig. 5a–c). Male and female SPs displayed distinct clustering patterns at both 3 and 28 months (Fig. 5a, b and Supplementary Fig. 5d). Clusters 0, 1, and 7 were more abundant in males, while clusters 2-5 were more abundant in females. Aging males exhibited a notable increase in cluster 0 and a decrease in cluster 1, while aging females displayed an increase in clusters 3–5 (Fig. 5b, c).

**Fig. 6:**
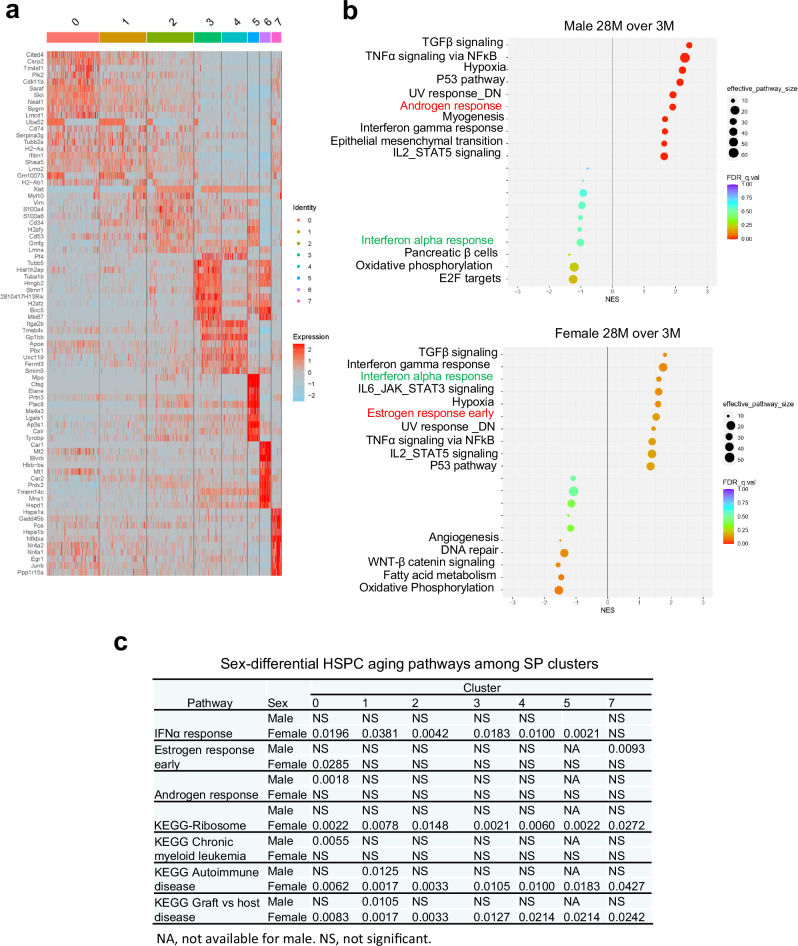
Sex-dependent differences in aging HSC gene expression signatures and signaling pathways. **a** Heatmap of the top ten genes in each cluster. **b** Age-related differential top Hallmark pathways of LT-HSCs (cluster 0) in males and females. **c** Sex-related differences in HSPC aging pathways among the SP clusters. The numbers shown are the *p* values of pathways enriched in old vs young mice in males and females.

Most of the SP cells were noncycling (Supplementary Fig. 6a). The Y-linked *Eif2s3y* gene expression marked male samples exclusively (Supplementary Fig. 6b). Slamf1 mRNA, which encodes CD150, showed indiscriminate expression in all clusters of both sexes (not shown), indicating that the protein, rather than the mRNA of CD150, has a distinguishing role in male and female SPs. Clusters were assigned to HSPC fractions based on gene expression profiles and characteristics (Figs. 5a, 6a and Supplementary Fig. 7). Cluster 0 was identified as LT-HSCs that were negative for the surface markers CD48 and Flt3 (Supplementary Fig. 7). Cluster 0 was more abundant in males than in females, regardless of age, increasing from 30% of total SP cells in young males to 63% in old males, while decreasing from 6% in young females to 4% in old females (Fig. 5b, c). Conversely, short-term (ST)-HSCs (cluster 1) decreased from 43% in young males to 17% in old males, suggesting a decline in the functional capacity of male HSCs to produce progeny, a trend not observed in old females (Fig. 5b, c). Cluster 2 comprised two subclusters: multipotent progenitors (MPPs) and common myeloid progenitors (CMPs), which was reduced in old females (Fig. 5c–e). Notably, old females had increased megakaryocyte progenitors (MkPs, cluster 3), megakaryocyte-erythroid progenitors (MEPs, cluster 4), and granulocyte-monocyte progenitors (GMPs, cluster 5). The increase in MEPs, MkPs and GMPs in old female SPs correlated with a more severe anemia phenotype and greater myeloid skewing observed in the long-lived females. Our findings demonstrate that SP cell increases in old males primarily stem from LT-HSC expansion, whereas the increase in SP cells in long-lived females results from committed progenitor expansion coupled with modest HSC increase.

Gene set enrichment analysis (GSEA) revealed that old male and female HSC cluster 0 shared several upregulated signaling pathways, including the TGFβ, TNFα, IL2-STAT5, IL6, interferon (IFN) γ, hypoxia, and p53 pathways (Fig. 6b and Supplementary Tables 1 and 2). These pathways are known to regulate normal mouse HSC functions and aging. [18, 42–50] Interestingly, the IFNα response was selectively activated in old female HSCs and other SP clusters (Fig. 6b, c). Additionally, the autoimmune disease, graft-versus-host disease (GvHD) and ribosome pathways were more selectively activated in the old female clusters (Fig. 6c). The activation of these pathways, especially the autoimmune and IFNα pathways, may be related to the hemolytic anemia observed in old females. Furthermore, the estrogen response and androgen response were selectively activated in cluster 0 in old females and males, respectively (Fig. 6c). Notably, unlike women experiencing menopause, female mice (C57BL/6 strain) undergo progressive reproductive senescence starting around 12 months of age [51], with plasma E2 levels decreasing to nearly ovariectomized levels by 24 months [52]. With low levels of circulating hormones in long-lived mice, these findings may suggest in situ hormone production or ligand-independent activation of these steroid receptors, as revealed in breast cancer tissues [53].

Bulk RNA-seq of SP cells confirmed scRNA-seq findings, revealing stark differences in aging between male and female cells. Differentially expressed genes (DEGs) associated with aging showed minimal overlap between sexes, while inter-sex DEGs overlapped significantly across age groups (Supplementary Fig. 8a, b). GSEA revealed similar pathways in male SP cell aging (Supplementary Fig. 8c and Supplementary Tables 3 and 4). Old females exhibited activated IFNα response, whereas old males showed a reduction. Old female SP cells enriched E2F targets, MYC targets, G2/M checkpoint, and oxidative phosphorylation, reflecting scRNA-seq clusters 3, 4, and 5 (Supplementary Tables 1 and 2). Male bulk RNA-seq revealed both androgen and estrogen signaling, mirroring scRNA-seq clusters 0 and 7 (Fig. 6c). In line with the changes in sex hormone signaling, the anti-androgen receptor (AR) signaling agent enzalutamide potently blocked methylcellulose colony formation by aging male HSPCs (Supplementary Fig. 9a), suggesting a crucial role of AR signaling in aging male HSC functions. This is in contrast to young male mouse HSCs on which castration has no significant impact [25]. The antiestrogen elacestrant moderately reduced methylcellulose colony formation by aging female HSPCs after second plating (Supplementary Fig. 9b), indicating that estrogen receptor signaling may still play a role in the maintenance of aging female HSCs, as observed in young mice [25]. Taken together, these data reveal sex-dependent differences in SP cell clustering and HSC gene expression in males and females during aging, which provides insight into their distinct blood cell differentiation phenotypes.

The scRNA-seq data could be analyzed by comparing HSC sex differences followed by age differences to address related but different questions (Supplementary Fig. 10a). No significant molecular differences were observed for LT-HSCs in young males vs females, whereas differences in ST-HSCs and some progenitors were observed (Supplementary Fig. 10b). Old females, however, exhibited enhanced IFNα and IFNγ responses, starting in LT-HSCs and amplifying in ST-HSCs. (Supplementary Fig. 10b). As in phenotypic analysis, this sex-based comparison, while informative, lacked the depth to uncover crucial age-related pathway changes specific to each sex. This is not unexpected as aging is the main driver of cellular abnormalities and diseases whereas sex plays an auxiliary role.

### Sex-dependent differences in HSC aging beyond BALB/c strain

**Fig. 7:**
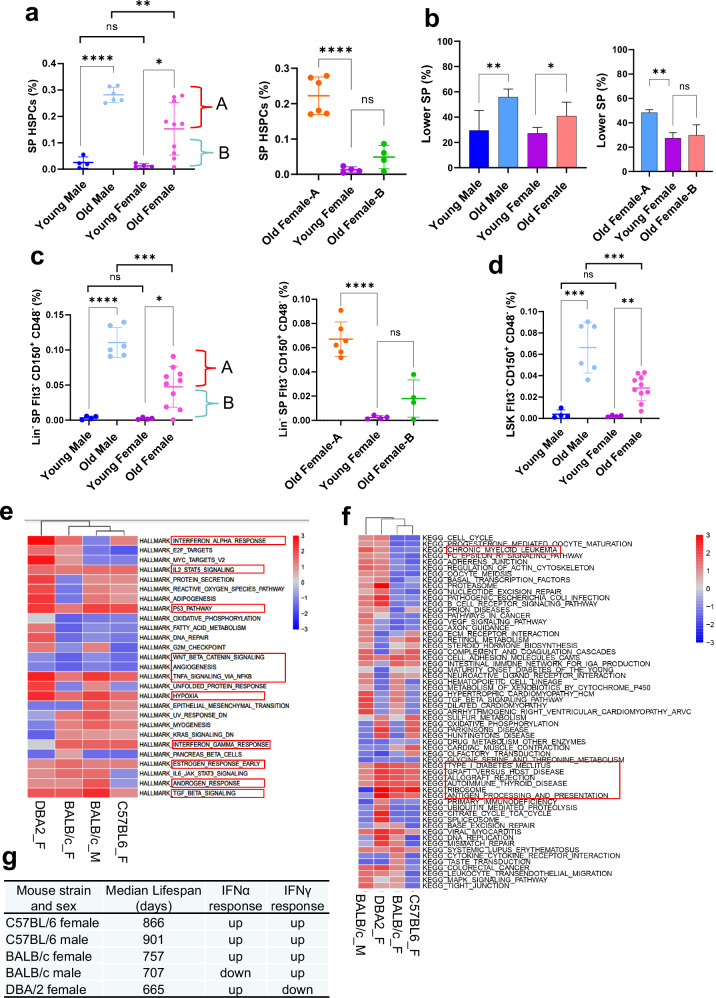
Comparison of HSC aging in different mouse strains. **a** SP HSPCs in 27 to 28-month-old C57BL/6 mice vs 3-month-old controls in two sexes. Old females were divided into two groups with HSPCs% above (A) and below (B) the average. **b** SP compartment analysis for lower SP fractions within total SP cells. **c** Frequencies of highly enriched HSCs based on SP separation. **d** Frequencies of highly enriched HSCs based on LSK separation. **e**, **f** Comparison of BALB/c scRNA-seq data of this study with GSE59114 dataset for aging C57BL/6 and DBA/2 females, which was analyzed directly using standard RNA-seq data analysis pipelines without reclassifying the cells. Cluster dendrograms for Hallmark (**e**) and KEGG (**f**) aging pathways of LT-HSCs from three mouse strains were shown. **g** IFNα/γ responses from LT-HSCs vs mouse lifespan. C57BL/6, a long-lived strain, activated IFNα and γ responses. DBA/2, shorter-lived, only triggered IFNα. BALB/c females outlived males, activating both responses compared to males’ sole IFNγ activation. *p < 0.05; **p < 0.01; ***p < 0.001; ****p < 0.0001; ns, not significant. The error bars represent one standard deviation.

To determine whether the observed sex-dependent differences in HSC aging were BALB/c strain specific, we examined HSCs in aged and young C57BL/6 mice of both sexes using SP- and LSK (Lin^-^Sca-1^+^c-Kit^+^)-based methods (Supplementary Fig. 11). Unlike BALB/c mice, old C57BL/6 mice didn’t undergo polarized SP changes in two sexes. SP was expanded in old C57BL/6 mice in both sexes, with old females exhibiting high variation (Fig. 7a). About 60% of old females (subgroup A) had increased SP, while 40% didn’t. This variability may stem from delayed HSC aging in females, as intrinsic SP changes were only observed in the subgroup A (Fig. 7b). LT-HSCs, enriched by Lin^-^SP Flt3^-^CD150^+^CD48^-^, expanded twice more in old males than females (Fig. 7c). LSK-based analysis confirmed this trend, with old males showing twice the expansion of old females (Fig. 7d). Therefore, the sex-dependent disparities in aging HSC expansion occur in both BALB/c and C57BL/6 strains, though more pronounced in BALB/c mice.

We cross-examined our scRNA-seq data with public scRNA-seq datasets on HSC aging in mice of known sex. Comparison with GSE59114 dataset [21] for aging C57BL/6 and DBA/2 females confirmed the common HSC aging pathways described above across three strains (Fig. 7e). Autoimmune-related pathways intensified in aged female HSCs universally (Fig. 7f). Incorporating another dataset (GSE147729) [54] for aging male HSCs maintained these trends, despite increased variability (Supplementary Fig. 12). Notably, IFNα/γ responses in LT-HSCs showed strain and sex differences correlating with lifespan, particularly in BALB/c mice (Fig. 7g). BALB/c LT-HSCs exhibited stronger androgen and estrogen responses compared to other strains. Aged BALB/c female HSCs uniquely displayed reduced adipogenesis, reactive oxygen species activity, and protein secretion (Fig. 7e). These factors may contribute to the pronounced sex-based differences observed in BALB/c mice.

### Sex impact on BCR-ABL1 transformation and CML development

We subsequently investigated the influence of sex on the transformation of aging HSCs by a human oncogene. Old male but not female BALB/c HSCs displayed an activated pathway for CML (Figs. 6c and 7f), a disease resulting from the BCR-ABL1-mediated transformation of HSCs [55]. CML is more prevalent in older men than women, and this sex difference becomes less prominent at younger ages [11, 12]. Mouse models are crucial for understanding CML and developing therapies; however, previous models were based on young mice. We have recently established the first aging mouse model of CML at 18 months of age [34], enabling us to examine the effects of sex on BCR-ABL1 transformation and CML progression in a context more reflective of elderly humans. Additionally, we showed that SIRT1 is activated by BCR-ABL1 transformation and that knockout of Sirt1 inhibits CML development in young mice [40, 56].

**Fig. 8:**
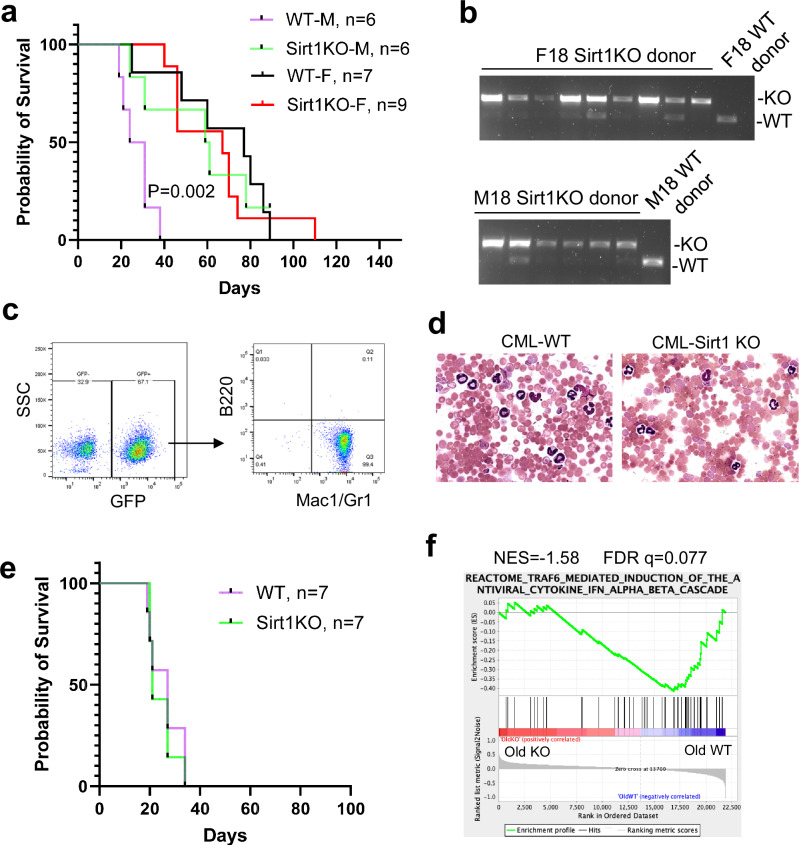
Differential impact of Sirt1 knockout on HSC transformation and CML development in aging male and female BALB/c mice. All the donors and recipients were 18 months old, and same-sex BMT was performed. **a** Survival curves of CML mice with a transduction rate (GFP^+^) of 10% for all groups of both sexes. Equal numbers of GFP^+^ BCR-ABL1-transduced donor cells were transplanted in all groups. **b** Confirming Sirt1^-/-^ donor cell genotypes in recipient mice by PCR genotyping of peripheral blood cells. **c** Flow cytometry analysis of a CML mouse blood. **d** Blood smears of CML mice developed from WT and Sirt1 knockout donor cells. **e** Comparison of survival curves with a transduction rate of 28% in females for both groups. **f** Sirt1 KO inhibited type I IFN induction in 18-month-old aging mouse SP cells, as determined by microarray gene expression analysis. WT wild type, KO knockout. F18 18-month-old female, M18 18-month-old male.

We performed BCR-ABL1 transformation of 18-month-old Sirt1^+/+^ and Sirt1^-/-^ donor cells and same-sex BMT in age-matched wildtype BABL/c recipients with an equal number of transduced cells, as described [34]. CML development was significantly faster in aging males than in aging females (Fig. 8a, b), mirroring the higher incidence in older men. Faster CML development in aging male mice than in aging females was confirmed in a separate experiment for tyrosine kinase inhibitor treatment (data not shown). Sirt1 knockout (KO) significantly inhibited CML development in aging males, as observed in young mice [40, 56], but unexpectedly had no effect on aging females (Fig. 8a, b). CML developed with both Sirt1^+/+^ and Sirt1^-/-^ donor cells showed similar phenotypes with marked expansion of neutrophils in the blood that were GFP^+^ and Mac1/Gr1^+^ (Fig. 8c, d) as described [34]. The lack of a Sirt1 KO effect in aging females was not due to the slower disease kinetics because increasing female BM cell transduction rates to accelerate CML development still failed to distinguish Sirt1^+/+^ and Sirt1^-/-^ CML development (Fig. 8e). These results contrast with young mice, where no sex-dependent disparities in CML development have been noticed with or without Sirt1 loss [40, 56], suggesting female HSC aging uniquely impacts Sirt1 KO influence on BCR-ABL1 transformation.

IFNα signaling was selectively activated in old female HSCs. IFNα was the standard of care for CML before the TKI imatinib [57]. IFNα can induce extremely stable event-free remission in some CML patients [58–60]. IFNα signaling activation in aging female HSCs may thus suppress the BCR-ABL1-mediated transformation of HSCs and CML development. SIRT1 is critical for the activation of type I IFN expression during infection [61]. Our microarray analysis of SP cells from 18-month-old Sirt1^-/-^ vs Sirt1^+/+^ mice [38] revealed that Sirt1 KO reduced type I IFN induction (Fig. 8f). Therefore, Sirt1 KO may paradoxically self-antagonize the inhibitory effect of Sirt1 loss on BCR-ABL1 transformation in aging females by decreasing IFNα signaling. The results demonstrated a sex-biased role of Sirt1 in the regulation of oncogenic transformation of HSCs in aging mice.

## Discussion

In this report, we described sex-dependent differences in HSC aging and leukemogenic potential in aging BALB/c mouse models. We demonstrated that myeloid-biased differentiation in aging males was driven by increasing myeloid cell output but by decreasing lymphoid cell output in aging females. Long-lived female BALB/c mice were more prone to developing hemolytic anemia and spontaneous myeloid leukemia (AML and B/myeloid MPAL). In contrast, BCR-ABL1 transformation in aging BALB/c mice led to faster CML development in aging males than in females. We showed that sex-dependent disparities in HSPC aging underlie these phenotypical differences between the two sexes. Male mice aged with a greater expansion of HSCs than females. BALB/c male and female LT-HSCs shared many aging pathways but differed in IFNα response and sex hormone signaling among others. These molecular differences also distinguished the BALB/c strain from C57BL/6 and DBA/2 strains, making BALB/c a better model for sex-differentiated hematological aging in humans.

Multiple factors may contribute to faster CML development in aging BALB/c males. First, the greater number of LT-HSCs in aging males may result in more transformed HSCs. Second, old female HSCs’ heightened IFNα signaling may inhibit BCR-ABL1 transformation. Third, the CML gene signature was detected in the old male LT-HSCs. These factors might explain sex differences in human CML. Conversely, long-lived female BALB/c mice developed AML or B/myeloid MPAL, mirroring AML trends in humans over 80. While AML generally affects men more [11, 12, 62], it’s more common in women past 80 [62]. This shift is often attributed to women’s longer lifespans, but chronic lymphocytic leukemia doesn’t show a similar pattern [63]. The mechanisms underlying the differences in leukemia types at different ages are still unclear, but the cell of origin for leukemias may provide an insight. CML stems from HSCs, while AML can originate from progenitors or HSCs [64–67]. More HSCs in aging males could yield more CML-initiating cells for faster CML development. In contrast, increasing progenitors from old females would make them more susceptible to AML/MPAL. Long-lived female mice showed expanded progenitor cells, potentially fueling AML or MPAL. Without BCR-ABL1 transformation, BMT with 110% lifespan BALB/c female donors produced AML or MPAL, but not with 75% lifespan donors as shown before [34]. Similarly, AML and MPAL have not been reported in HSC aging studies in C57BL/6 mice where BMT donors are generally under 24 months ( < 80% lifespan) [22]. Whether C57BL/6 donor mice at 110% lifespan would produce AML/MPAL phenotypes is unknown. But strain differences may also play a role, as BALB/c mice harbor a hypomorphic p16^INK4a^ allele [28, 29], possibly increasing leukemia susceptibility.

IFNα/γ responses in old LT-HSCs correlated with mouse lifespan. Old BALB/c males showed reduced IFNα response, mirroring their shorter lifespans. IFNα can stimulate HSC proliferation and promote HSC functional attrition [42, 68]. Extended female mouse lifespan occurred at the expense of enhanced myeloid skewing and hemolytic anemia in old females. This aligns with women’s better survival during viral infections like COVID-19 [69–71], yet higher susceptibility to autoimmune diseases [2, 72] and that female bone marrow donor cells trigger more GvHD in male recipients [73–75]. Thus, sex-dependent disparities in HSC aging may influence these disorders and warrant further research.

Sirt1 knockout unexpectedly hindered CML development in aging male mice, but not females. This sex disparity may stem from IFNα response differences. IFNα signaling decreases in human CML leukemia stem cells (LSCs) [76], and some patients respond well to IFNα therapy [58–60]; however, the mechanisms of IFNα action in CML remain elusive. Increased IFNα response in aging female HSCs could reduce CML-initiating cell potency. Conversely, diminished HSC stemness might impede transformation into potent CML LSCs. Both scenarios could weaken CML activity. Sirt1 knockout may negate IFNα‘s suppressive effects in aging females, counteracting CML inhibition through other pathways [40, 56, 77]. The intricate interplay between Sirt1 knockout and IFNα signaling in aging mice with CML warrants further investigation.

In conclusion, we demonstrate that sex shapes HSC aging and leukemia development differently in males and females. Our mouse models reveal patterns mirroring human sex disparities in anemia and myeloid leukemia with age. Female HSCs show less clonal expansion than males as they age, while committed progenitors expand more in aging females. Sex-dependent molecular differences of HSC aging underlie these phenotypic disparities. These novel insights into sex-differential HSC aging could inform targeted approaches for treating age-related blood disorders in each sex.

## Materials and methods

Animal studies were approved by the City of Hope Institutional Animal Care and Use Committee. The details regarding animal housing, breeding, genotyping, blood cell count, SP analysis, bone marrow transplantation, CML mouse model, and histological analysis are provided in the Supplementary Methods. Briefly, BALB/c (Taconic) and Sirt1 knockout mice in this strain were bred and aged in house as described previously [34]. Aged C57BL/6 mice were ordered from National Institute of Aging aged rodent colonies and young C57BL/6 from Jax Mice. For transplantation with aging BALB/c BM cells, equal number of unfractionated BM nucleated cells (three to five millions per mouse) were transplanted into lethally irradiated recipients by retro-orbital injection. For CML studies, the BCR-ABL1 transduced lineage-depleted cells with an equal percentage of GFP^+^ cells in 0.4 million total cells/mouse were transplanted. For SP analysis, BM nucleated cells were labeled with Hoechst 33342, followed by lineage depletion, and then surface marker staining for flow cytometry. Anemia was defined as a hemoglobin concentration more than 2 standard deviations below the mean of 3-month-old baseline hemoglobin values [78].

The details of scRNA-seq and RNA-seq were provided in the Supplementary Methods. Briefly, BM SP cells were fresh isolated from 2 to 4 mice per group, and at least 5000 SP cells were collected for each sample. About 1000 cells were captured per sample on a 10xGenomics Chromium controller using a 10X V3.1 Single Cell 3’ Solution kit. The libraries were prepared and sequenced with the paired end setting on Illumina NovaSeq 6000 platform with a depth of 100 –135 K reads per cell. Raw sequencing data were processed and uploaded to R using the Seurat package. Uniform Manifold Approximation and Projection (UMAP) coordinates [79] were used to visualize the resulting clusters. Pathway analysis was performed by GSEA 4.0.3 in Hallmark and KEGG terms. The leftover cells from samples prepared for scRNA-seq were used for bulk RNA-seq and the libraries were prepared with SMART-Seq® Ultra Low Input RNA Kit. Sequencing was performed on Illumina HiSeq 2500 with the single read mode. After filtration, 11,673 genes out of 22,850 genes with RPKM ≥ 1 in at least one sample were used to generate hierarchical clustering plot by CLUSTER 3.0. DEG were identified by edgeR (v.3.20.9) and hierarchical clustering heatmap for DEG was generated. Pathway changes were analyzed by GSEA.

For animal transplantation studies, Kaplan‒Meier survival analysis was performed, and statistical significance was calculated using the log-rank test. The two-tailed Student’s *t*-test was performed for other data analyses except for anemia where one-tailed *t*-test was used for analyzing hemoglobin reduction. *P* < 0.05 was considered statistically significant. Error bars are shown with standard deviations. All the measurements were taken from distinct samples.

## Supplementary information

Supplementary Methods

Supplementary Figures

Supplementary Table 1

Supplementary Table 2

Supplementary Table 3

Supplementary Table 4

## Data Availability

The raw scRNA-seq and bulk RNA-seq data reported in this paper have been deposited as SuperSeries files in GEO with the accession code GSE262181 (scRNA-seq subseries GSE262145 and bulk RNA-seq subseries GSE262180). The GSEA reports of the scRNA-seq and RNA-seq data are provided in Supplementary Tables 1–4. Any remaining information can be obtained from the corresponding author upon reasonable request.
