# Supplementary material for: Sex-dependent differences in hematopoietic stem cell aging and leukemogenic potential
Source: Oncogene. 2024 Nov 1;44(2):64–78. doi: 10.1038/s41388-024-03197-9 (PMC11706783; doi:10.1038/s41388-024-03197-9)
Supplement: Supplementary file 1 — Supplementary Methods [file 41388_2024_3197_MOESM1_ESM.pdf]

Sex-dependent differences in hematopoietic stem cell aging and leukemogenic potential

Chunxiao Zhang,[1,‡,⁋] Taisen Hao,[1,ǂ,⁋] Alessia Bortoluzzi,[1] Min-Hsuan Chen,[2] Xiwei Wu,[2] Jinhui Wang,[2] Richard Ermel,[3] Young Kim,[4] Shiuan Chen[1] and WenYong Chen[1 *]

SUPPLEMENTARY METHODS

Mice

Animal studies were conducted under an approved protocol by City of Hope Institutional Animal Care and Use Committee. All mice were housed and maintained in accordance with the Guide for the Care and Use of Laboratory Animals at the AAALAC International accredited specific-pathogen-free Animal Resources Center within the Center for Comparative Medicine at City of Hope and fed ad libitum with LabDiet 5053 standard mouse chow and provided reverse-osmosis treated water. The animal room temperature was held within a range of 68 ºF to 75 ºF, humidity was 30% to 70%, and light cycle was 12 hours light:12 hours dark. Mice were routinely attended to by animal health technicians and veterinarians. BALB/c (Taconic strain) were bred and aged in house. Aged (27 to 28 months) C57BL/6 mice were ordered from National Institute of Aging aged rodent colonies and young (3 months) C57BL/6 from Jax Mice. Aging BALB/c mice may be affected by several non-neoplastic and neoplastic conditions. Generally, mice with visible or palpable tumors, open wounds, severe ulceration and inflammation, abnormal breathing, and unusual body postures were excluded from the study according to the established criteria as described previously.[1] Mice used for single cell sequencing and donors of bone marrow transplant were necropsied after euthanasia to exclude the ones with obvious abnormal organs such as enlarged spleen, enlarged liver, lung tumor, endometrial cancer, or prostate tumor. The SIRT1

knockout mice used in the study were maintained as reported previously.[2] Briefly, SIRT1 knockout mice were backcrossed to the BALB/c strain (Taconic) for at least nine generations, and SIRT1$^{+/-}$ mice were bred each other to produce SIRT1$^{-/-}$ mice using a consecutive fostering protocol. Mice were genotyped with the following primer set: Forward primer: CTTGCACTTCAAGGGACCAA, Reverse primer -1: GTATACCCACATCTGAG, Reverse primer -2: CTACCACTCCTGGCTACCAA. All three primers were mixed to perform PCR to obtain a band of ~800 bp for the KO allele and a band of ~500bp for the WT allele.

Blood cell counts

Mouse peripheral blood was collected by tail snipping and differential blood cell counts were analyzed by IDEXX ProCyte Dx Hematology Analyzer. Variation of date of birth of individual mice at each age group (3, 12, 18 and 28 months) was limited to less than 2 weeks at the time of experimentation. Anemia was defined as a hemoglobin concentration more than 2 standard deviations below the mean of 3-month-old baseline hemoglobin values.[3]

Bone marrow harvesting and processing

Femurs and tibiae were dissected and removed of surrounding tissue. Bone marrow (BM) was flushed out into a 50 ml conical tube with cell suspension medium (RPMI 1640) using a 27G needle and a 10 mL syringe, and the released BM was triturated to a homogeneous mixture by a syringe with a 22G needle. Dissociated cells were filtered through a 40-µm cell strainer, spun down at 1000 r.p.m. for 5min at 4°C and then incubated with 2ml ACK lysis buffer (Gibco) for 3min at room temperature for lysis of red blood cells. The cells were neutralized with 15ml cell

suspension medium, spun down and re-suspended in appropriate buffer for further steps. The cells were defined as total (nucleated) BM cells at this point.

HSC purification and analysis

For side population, total (nucleated) BM cells were stained with 5 µg/mL Hoechst 33342 at 37 ºC in the DMEM+ media (DMEM with 2% heat inactivated fetal bovine serum and 1 mM HEPES) in a density of 1 million cells per mL for 90 min exactly. Cells were spun down, washed once by ice cold phosphate-buffered saline (PBS)-BSA buffer (PBS + 0.05% BSA) and resuspended in PBS-BSA buffer. Cells were always put on ice during the following steps. Lineage depletion was then performed for the Hoechst-stained BM cells using EasySep Stem Cell Enrichment Kit (StemCell Technologies) according to the manufacture's recommendations. The enriched BM cells were then stained for 20 minutes with the PE-conjugated lineage antibody cocktail (Gr-1, clone RB6-8C5; CD11b/Mac-1, clone M 1/70; B220, clone RA3-6B2; Ter119, clone TER-199; and CD3e, clone 145-2C11. They were from BD Biosciences) and other cell markers. The PE-Lin/SP/APC-CD150 combination was used to analyze HSCs by flow cytometry as described [4]. For LT-HSC analysis, the cells were stained for 20 minutes with FITC-conjugated CD117 (clone 2B8), BV421- conjugated Sca-1 (clone D7), APC-conjugated CD150 (cloneTC15-12F12.2), PE/Cy7-conjugated CD48 (clone HM48-1), PerCP-eFluor 710 (clone A2F10)-conjugated Flt3. All these antibodies were from BD Biosciences.

Bone marrow transplantation and aging mouse model of CML

For transplantation with aging BM cells, recipient mice were placed under Sulfatrim antibiotic diet at Day 1 and remained on this diet for at least two months. Lethal irradiation for recipients was

performed at Day 4 with 2 doses of 450 Rad radiation (900 Rads in total) separated by at least 3 hours. Three to five million BM cells were transplanted into each mouse by retro-orbital injection. Mouse health was closely monitored, and mice were euthanized according to the American Veterinary Medical Association Guidelines. SRY genotyping was performed to track donor cells. Genomic DNA was extracted from blood mononuclear cells using DNeasy blood & tissue kit (QIAGEN). About 100 ng DNA per reaction was used for PCR as described.[5] For Xist genotyping, RNA was extracted using Directzol RNA Microprep Kit (Zymo Research), and the first strand DNA was synthesized with Superscript III kit (Invitrogen), and Xist gene expression was analyzed by using SYBR Green qPCR SuperMix kit (KAPA biosystems). β-actin was used as the housekeeping gene control.

For the aging mouse model of CML, a newly modified protocol for bone marrow transduction and transplantation was conducted as described[1]. Briefly, lineage-depleted total BM cells were cultured in stimulation medium (RPMI 1640, 20% FBS, 1% penicillin/streptomycin, 20ng/ml of recombinant murine IL-3, 20ng/ml of IL-6, and 60ng/ml of SCF [Peprotech]) at 1 million cells per milliliter at Day 1. Two rounds of cosedimentation retroviral transduction as described[6] were performed at Day 2 and Day 3 individually in 24-well plates which were coated with Retronectin [10 μg/well (Takara Bio)] at Day 1. For each round, fresh virus carrying ecotropic BCR-ABL MIG210 with a titer $\geq 2x10^6$/ml, which was packaged from Phoenix-Eco cells, was used for transduction. Recipient mice that were pre-conditioned by Sulfatrim antibiotic as described above were lethally irradiated, and the transduced cells were transplanted through retro-orbital injection right after the second dose of irradiation, with 0.4 million cells/mouse unless indicated. CML from

donor cells was confirmed by GFP expression and in the case of Sirt1 knockout, the cells were further confirmed by Sirt1 genotyping.

scRNAseq analysis

For each age group, BM was harvested from 2 to 4 mice of the same age group. BM cell SP staining and purification were processed as described above. After staining with the PE-conjugated lineage antibody cocktail, the cells were washed twice with PBS-BSA buffer and resuspended in 500µl PBS. SP cells were immediately sorted into 400 µl DMEM containing 0.04% BSA with an Aria SORP cell sorter, and at least 5,000 SP cells were collected for each sample. The cell numbers and viability were confirmed by both automated cell counter and manual counting. The cells viability of all samples we processed were greater than 80%. About 1,000 cells were captured per sample on a 10xGenomics Chromium controller using a 10X V3.1 Single Cell 3' Solution kit (10xGenomics, Chromium Next GEM Single Cell 3' Regent kits V3.1, Cat. PN-1000268). All protocols were performed following the manufacture's instruction. Final sequencing libraries were analyzed on a High Sensitivity DNA Chip (Agilent, Cat 5067-4626) to determine the library size. The library concentration was determined with a Qubit high Sensitivity DNA assay Kit (Thermo, Cat. Q32854). The libraries were sequenced with the paired end setting of 28 cycles of read1, 101 cycles of R2, 8 cycles of index i7 and 8 cycles of index i5 read on Illumina NovaSeq 6000 platform with S4 Reagent kit v1.5 (Illumina, Cat 20028313) at TGEN with a depth of 100K - 135K reads per cell.

Raw sequencing data were processed using the 10× Genomics Cell Ranger pipeline (version 3.1.0) to generate FASTQ files and aligned to mm 10 genome to generate gene expression counts. The

digital expression matrices for cells passed initial quality control were uploaded to R (3.6.1) using the Seurat package (3.1.3.9002)[7] for the comprehensive downstream analyses and visualization. The cells were further filtered to remove potential empty droplets (<1000 UMI) and doublets (>9000 UMI), and low-quality cells with mitochondrial RNA read rate > 15%. Information of cell counts, genes and UMI per cells of individual samples after normalization were shown in Supplementary Fig. 5. Uniform Manifold Approximation and Projection (UMAP) coordinates were used to visualize the resulting clusters. Normalized and scaled data were clustered using the 14 top significant principal components of highly variable genes (HVG) with a resolution of 0.6. Top 10 cluster-specific genes were identified to generate heatmap, and selected markers were used to generate feature plots for identified cell clusters. Pathway analysis was performed by Gene set enrichment analysis (GSEA 4.0.3) in Hallmark and KEGG terms. Pseudotime trajectory analysis was performed using the Monocle2 package (2.12.0) in R after converting Seurat objects into CellDataSet format.

RNAseq analysis

The leftover cells from samples prepared for scRNAseq were used for bulk RNAseq purpose, and the libraries were prepared with SMART-Seq® Ultra Low Input RNA Kit for Sequencing – v4 (TaKaRa, Cat. 634888) according to the manufacturer's protocol. The final libraries were validated with the Agilent Bioanalyzer DNA High Sensitivity Kit and quantified with Qubit. Sequencing was performed on Illumina HiSeq 2500 with the single read mode of 51cycle. Real-time analysis (RTA) 2.2.38 software was used to process the image analysis. Raw FASTQ data files were pre-processed under adapter trimming by Trimmomatic (v.0.38) and polyA removal by FASTP (v.0.19.4). After reads were filtered for quality control by RSeQC (v.2.5), 11673 genes out of

22,850 genes with RPKM ≥ 1 in at least one sample were used to generate hierarchical clustering plot by CLUSTER 3.0. Differentially expressed genes (DEG) were identified by edgeR (v.3.20.9). Up-regulated DE genes were defined as fold-change ≥ 1.5 while fold-change ≤0.7 were defined as down-regulated DE genes. 3361 DE genes were also used to generate hierarchical clustering heatmap by edgeR (v.3.20.9). GSEA were analyzed and compared among samples against databases h.all.v7.2.symbols.gmt and c2.cp.kegg.v7.2.symbols.gmt with FDR < 0.25 by GSEA (v. 4.0.3).

Flow cytometry analysis

Lineage analysis for blood, bone marrow and spleen cells were performed by staining the nucleated cells with an antibody cocktail containing PE-Cy7 labeled anti- CD11b and anti-Gr1 for myeloid cells, APC labeled anti-B220 for B cells and BV605 labeled anti-CD3e for T cells. BCR-ABL expression in CML mouse samples was monitored by GFP reporter using flow cytometry analysis. Data were collected on BD LSR Fortessa at the City of Hope Analytic Cytometry Core and analyzed with FlowJo software.

Histological analysis

Blood film slides were made at the lab and H&E staining was performed with a Hemacolor (Sigma Aldrich) rapid staining of blood smear kit. Mouse tissues were fixed in 10% formalin and submitted to the City of Hope Pathology Core for H&E staining. All images were taken with a Nikon Biostation at the Light Microscopy Core of City of Hope.

Statistics and Reproducibility

For animal transplantation studies, Kaplan-Meier survival analysis was performed and statistical significance was calculated using the log-rank test. The two-tailed Student's *t*-test was performed for other data analyses except for anemia where one-tailed *t*-test was used for analyzing hemoglobin reduction. $P < 0.05$ was considered statistically significant. Error bars were shown with standard deviation. All measurements were taken from distinct samples.

References:

1. Hao, T., *et al.* An aging mouse model of human chronic myeloid leukemia. *Oncogene* **40**, 3152-3163 (2021).
2. Wang, Z., Chen, C.C. & Chen, W.Y. CD150- Side Population Defines Leukemia Stem Cells in a BALB/c Mouse Model of CML and Is Depleted by Genetic Loss of SIRT1. *Stem Cells* **33**, 3437-3451 (2015).
3. Raabe, B.M., Artwohl, J.E., Purcell, J.E., Lovaglio, J. & Fortman, J.D. Effects of weekly blood collection in C57BL/6 mice. *Journal of the American Association for Laboratory Animal Science : JAALAS* **50**, 680-685 (2011).
4. Weksberg, D.C., Chambers, S.M., Boles, N.C. & Goodell, M.A. CD150- side population cells represent a functionally distinct population of long-term hematopoietic stem cells. *Blood* **111**, 2444-2451 (2008).
5. Wang, Z., *et al.* Loss of SIRT1 inhibits hematopoietic stem cell aging and age-dependent mixed phenotype acute leukemia. *Communications biology* **5**, 396 (2022).
6. Yuan, H., *et al.* Activation of stress response gene SIRT1 by BCR-ABL promotes leukemogenesis. *Blood* **119**, 1904-1914 (2012).
7. Butler, A., Hoffman, P., Smibert, P., Papalexi, E. & Satija, R. Integrating single-cell transcriptomic data across different conditions, technologies, and species. *Nat Biotechnol* **36**, 411-420 (2018).
