# Supplementary material for: Sex-dependent differences in hematopoietic stem cell aging and leukemogenic potential
Source: Oncogene. 2024 Nov 1;44(2):64–78. doi: 10.1038/s41388-024-03197-9 (PMC11706783; doi:10.1038/s41388-024-03197-9)
Supplement: Supplementary file 2 — Supplementary Figures [file 41388_2024_3197_MOESM2_ESM.pdf]

Sex-dependent differences in hematopoietic stem cell aging and leukemogenic potential

Chunxiao Zhang,[1,ⱡ,⁋] Taisen Hao,[1,ǂ,⁋] Alessia Bortoluzzi,[1] Min-Hsuan Chen,[2] Xiwei Wu,[2]
Jinhui Wang,[2] Richard Ermel,[3] Young Kim,[4] Shiuan Chen[1] and WenYong Chen[1 *]

List of Supplementary Figures

Supplementary Fig. 1 Comparison of hematological phenotypes between the two sexes at each time point.

Supplementary Fig. 2 The same-sex BMT of long-lived donor mouse BM to young recipients.

Supplementary Fig. 3 The cross-sex BMT of long-lived donor mouse BM to young recipients.

Supplementary Fig. 4 Bone marrow histology of young and healthy long-lived BALB/c mice.

Supplementary Fig. 5 scRNAseq of male and female, young and old mouse SP cells.

Supplementary Fig. 6 Gene expression in UMAP clusters of SP HSPCs

Supplementary Fig. 7 HSC marker CD48 and Flt3 gene expression in SP clusters.

Supplementary Fig. 8 Bulk RNAseq analysis of SP HSPCs in males vs females.

Supplementary Fig. 9 Effects of blocking sex hormone signaling on aging HSPCs.

Supplementary Fig. 10 Analysis of sex and age differences of HSCs by different comparisons.

Supplementary Fig. 11 HSC analysis in C57BL/6 mice.

Supplementary Fig. 12 Comparison of HSC aging pathways in different mouse strains.

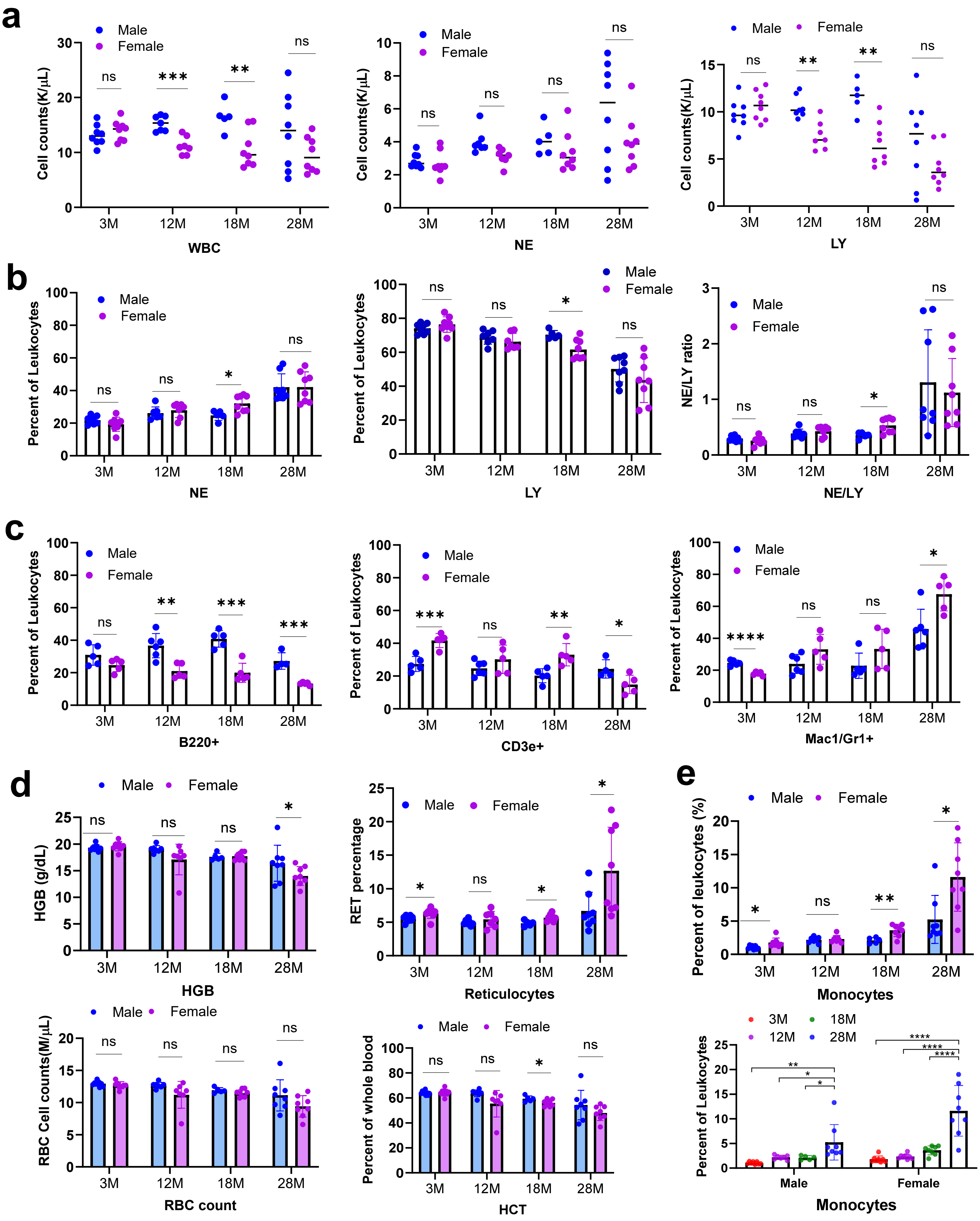

Supplementary Fig. 1 Comparison of hematological phenotypes between the two sexes at each time point.

(a-d) The data from Fig. 1 were re-plotted for two sex comparison for each time point. **(e)** Comparison of blood monocyte counts.

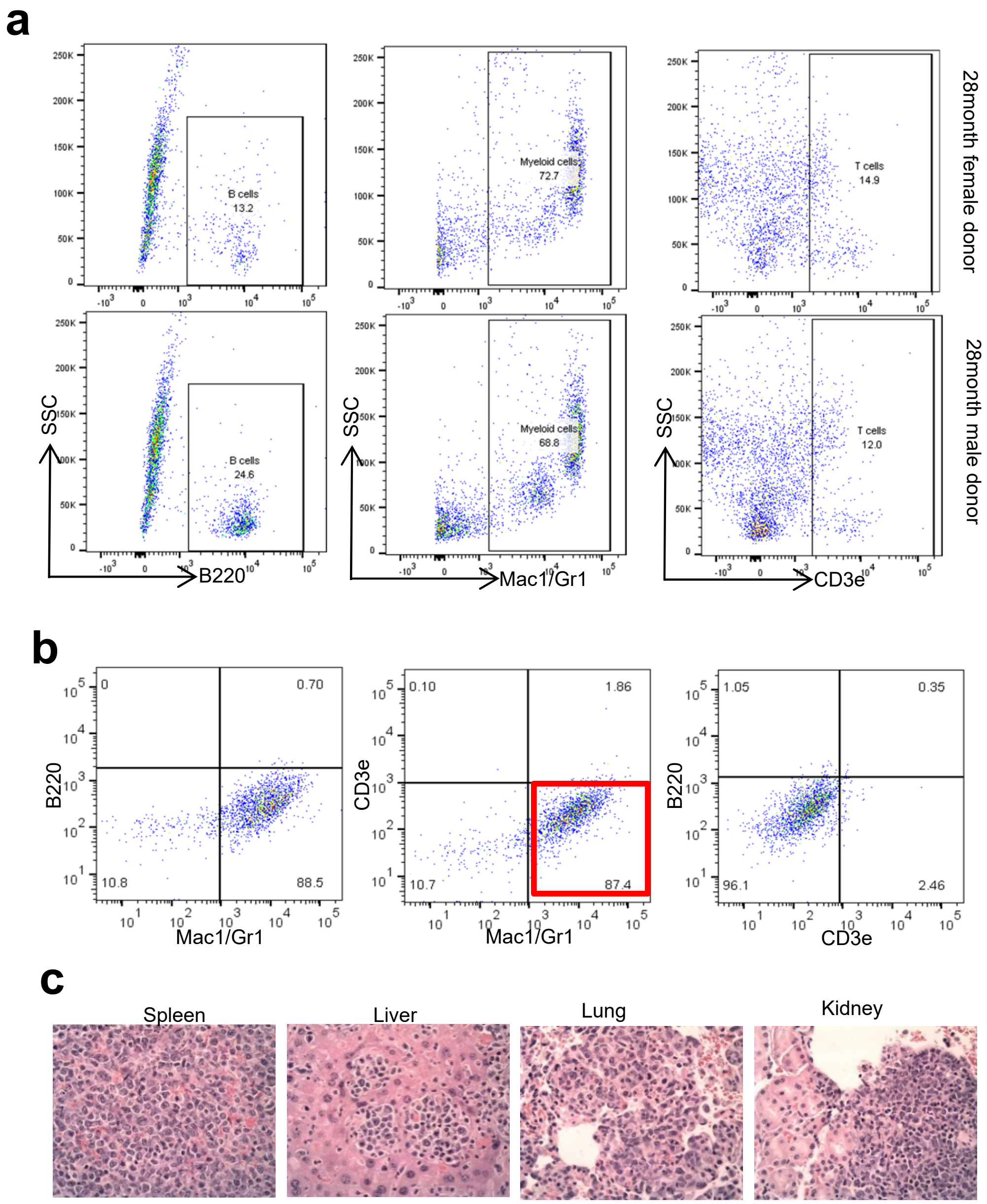

Supplementary Fig. 2 The same-sex BMT of long-lived donor mouse BM to young recipients.

(a) Flow cytometry analysis of long-lived male and female donor BM cells showing comparable profiles. **(b, c)** Analysis of the first deceased female recipient mouse by flow cytometry of splenic cells (b) and histology of tissue sections (c), both showing massive myeloid leukemia cell infiltration.

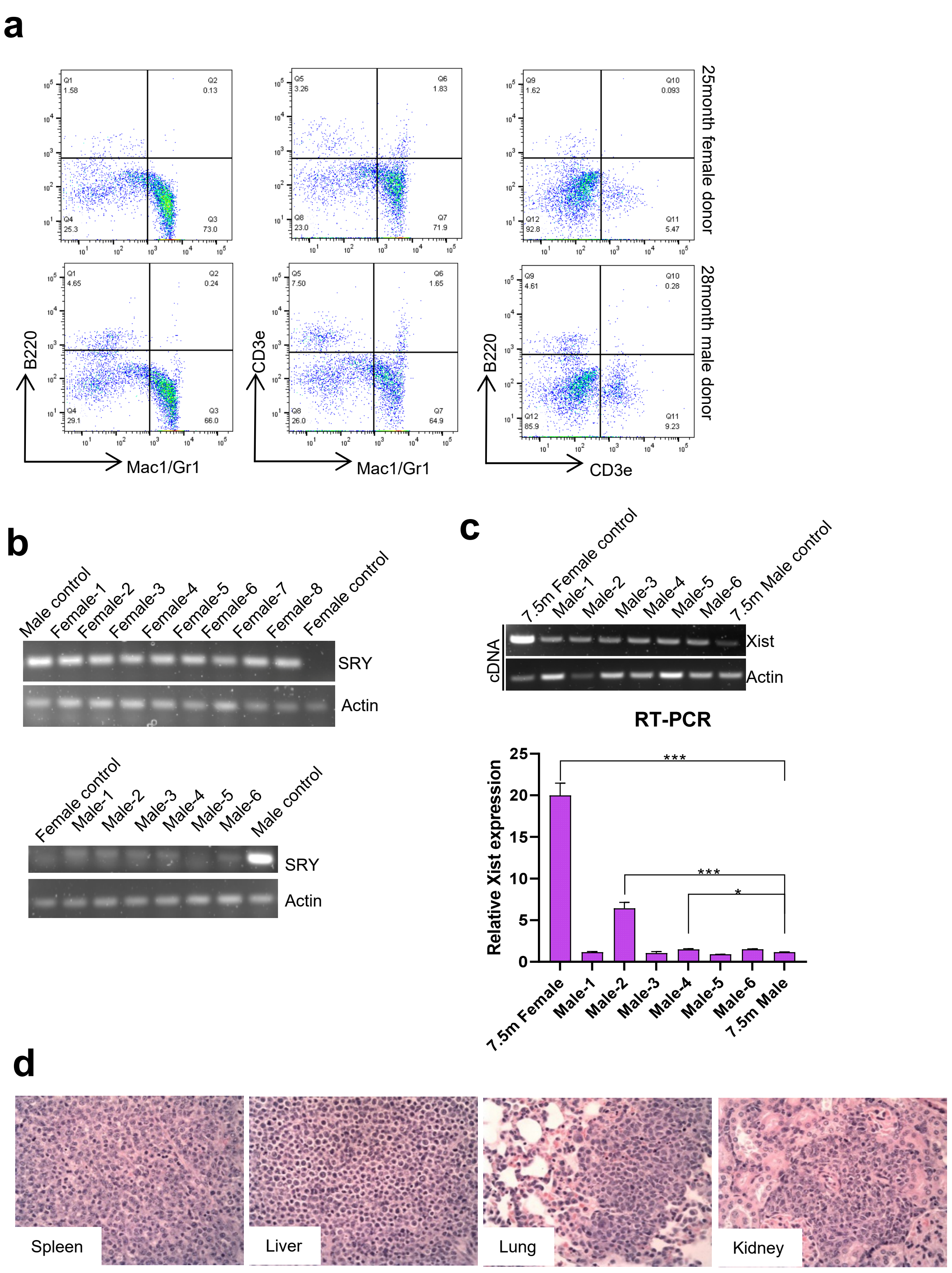

Supplementary Fig. 3 The cross-sex BMT of long-lived donor mouse BM to young recipients. **(a)** Flow cytometry analysis of 28-month male and 25-month female donor BM cells for the cross-sex BMT showing comparable profiles. **(b)** SRY genotyping of BMT recipient mouse genomic DNA from blood. **(c)** Analysis of Xist gene expression in blood cells by RT-qPCR of OF-YM BMT recipients. Xist expression was significantly reduced in all recipients, compared to blood cells from a 7.5-month-old female control. **(d)** Tissue sections of a recipient mouse with MPAL showing in Fig. 2f,g.

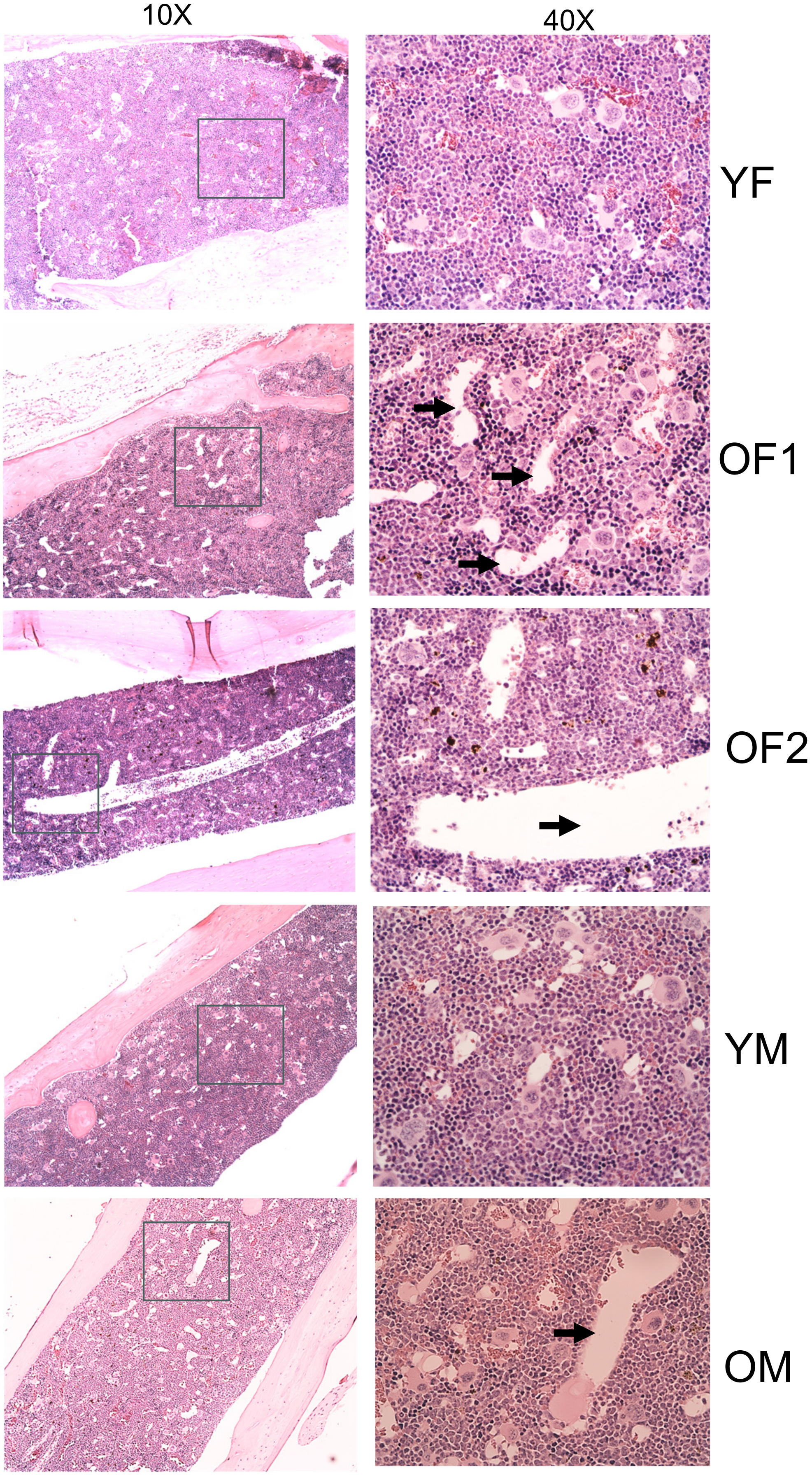

Supplementary Fig. 4 Bone marrow histology of young and healthy long-lived BALB/c mice. Enlarged sinusoids in old female 1 and 2 (OF1 and OF2) and old male (OM) mice were indicated by arrows as compared to young female and male (YF And YM) mice. Squares are the areas imaged with higher magnification.

a

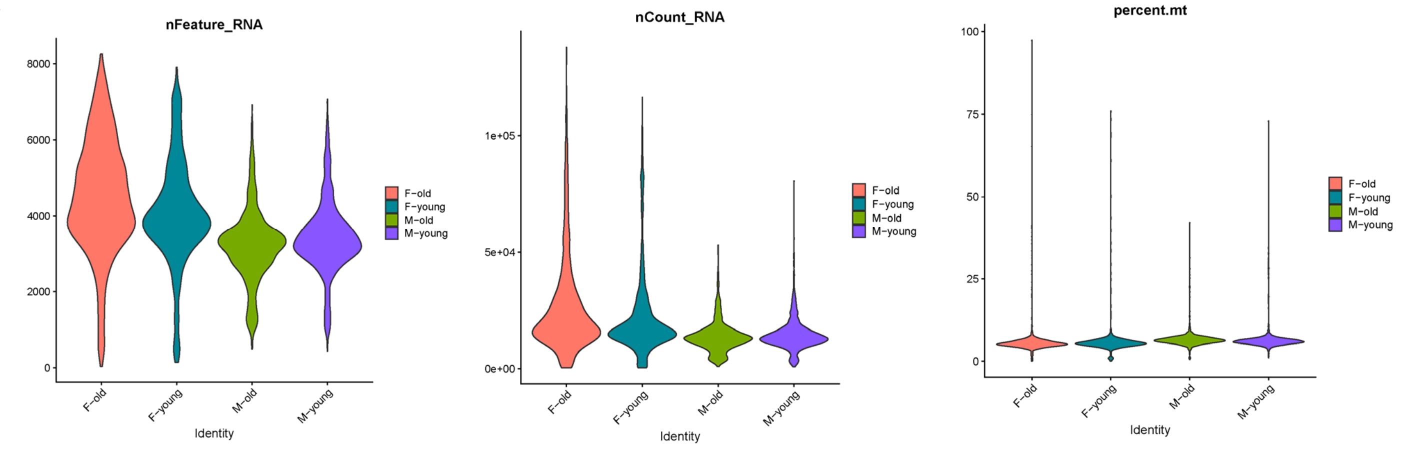

b

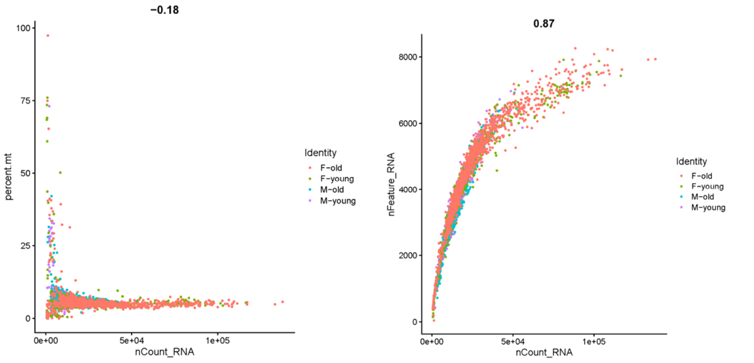

c

sample	cell #	reads (million)	Mean reads per cell (K)	Median genes per cell
**Male_3M**	1,035	121	117	3342
**Male_28M**	814	100	122	3230
**female-3M**	830	105	123	3944
**female-28M**	1,295	181	134	4443

d

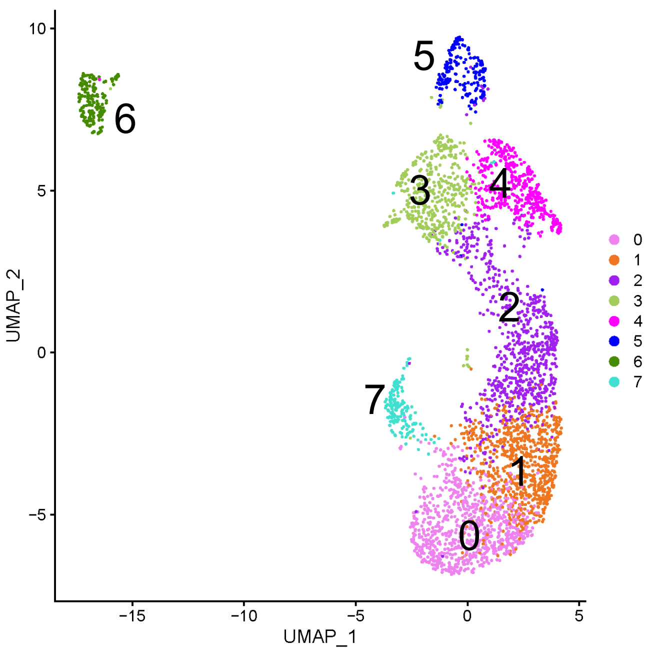

Supplementary Fig. 5 scRNAseq of male and female, young and old mouse SP cells. **(a)** Quality control profiles of scRNAseq of mouse SP. All samples displayed consistent good quality. **(b)** Feature scatter plots. The cells were filtered to remove potential empty droplets (<1000 UMI) and doublets (>9000 UMI). Cells with less than 200 genes detectable and >15% mitochondria content were removed. **(c)** Cell number and reads for each sample group. **(d)** UMAP of all clusters of SP cells in the aggregate.

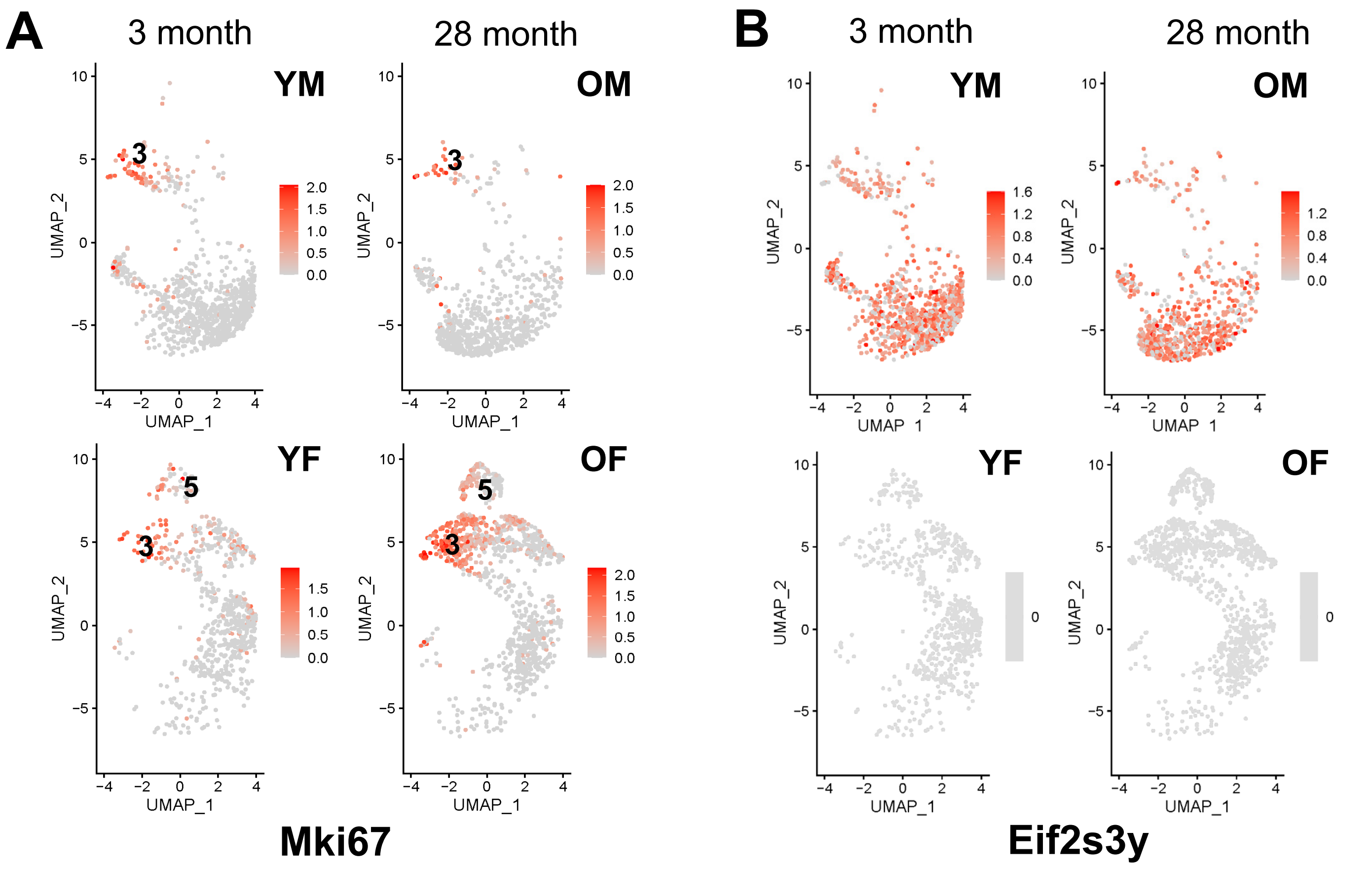

Supplementary Fig. 6 Gene expression in UMAP clusters of SP HSPCs. **(a)** Cell cycle status by Mki67 expression. **(b)** Sex difference confirmed by the Y-linked Eif2s3y expression. YM, young males; OM, old males; YF, young females; OF, old females.

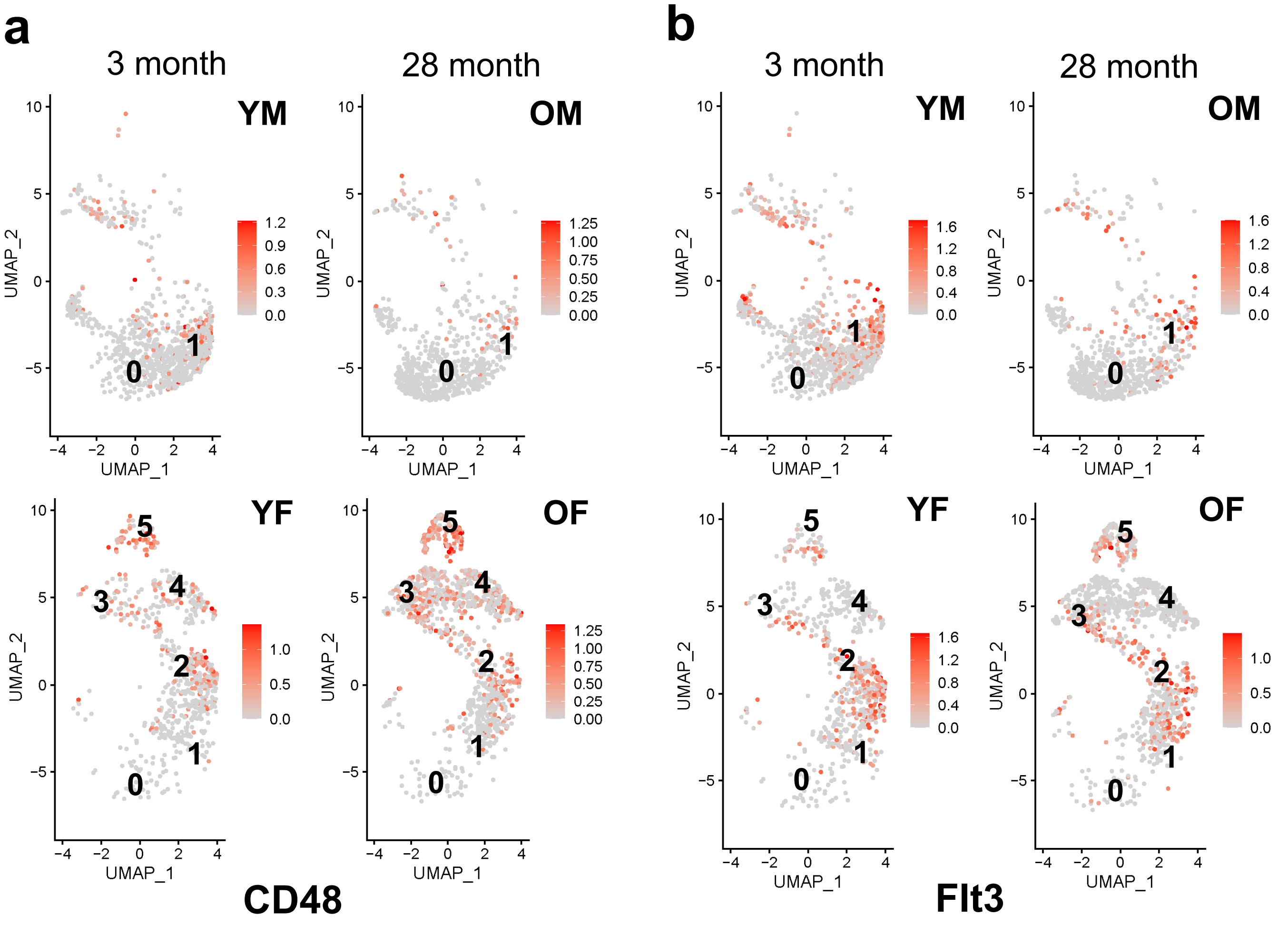

Supplementary Fig. 7 HSC marker CD48 and Flt3 gene expression in SP clusters.

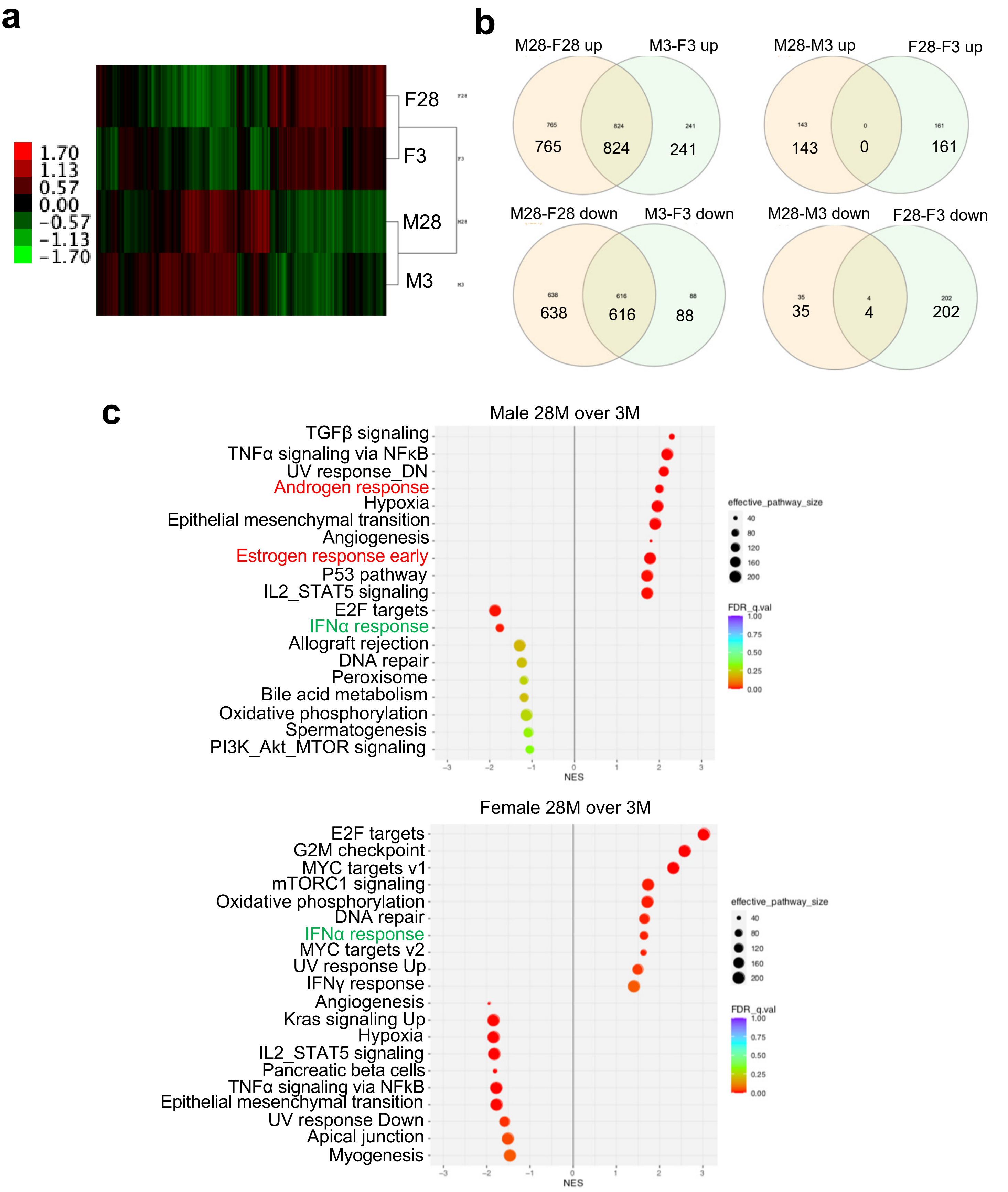

Supplementary Fig. 8 Bulk RNAseq analysis of SP HSPCs in males vs females.
(a) The hierarchical clustering of 11,673 genes out of the 22,850 genes with RPKM >= 1 in at least one sample. F28 and F3: female 28 and 3 months; M28 and M3: male 28 and 3 months. **(b)** Venn diagram analysis of gene changes in mice with the same age and different sexes (left) or the same sex and different ages (right). **(c)** GSEA analysis of bulk RNAseq of SP cells for leading Hallmark pathway changes over aging in two sexes.

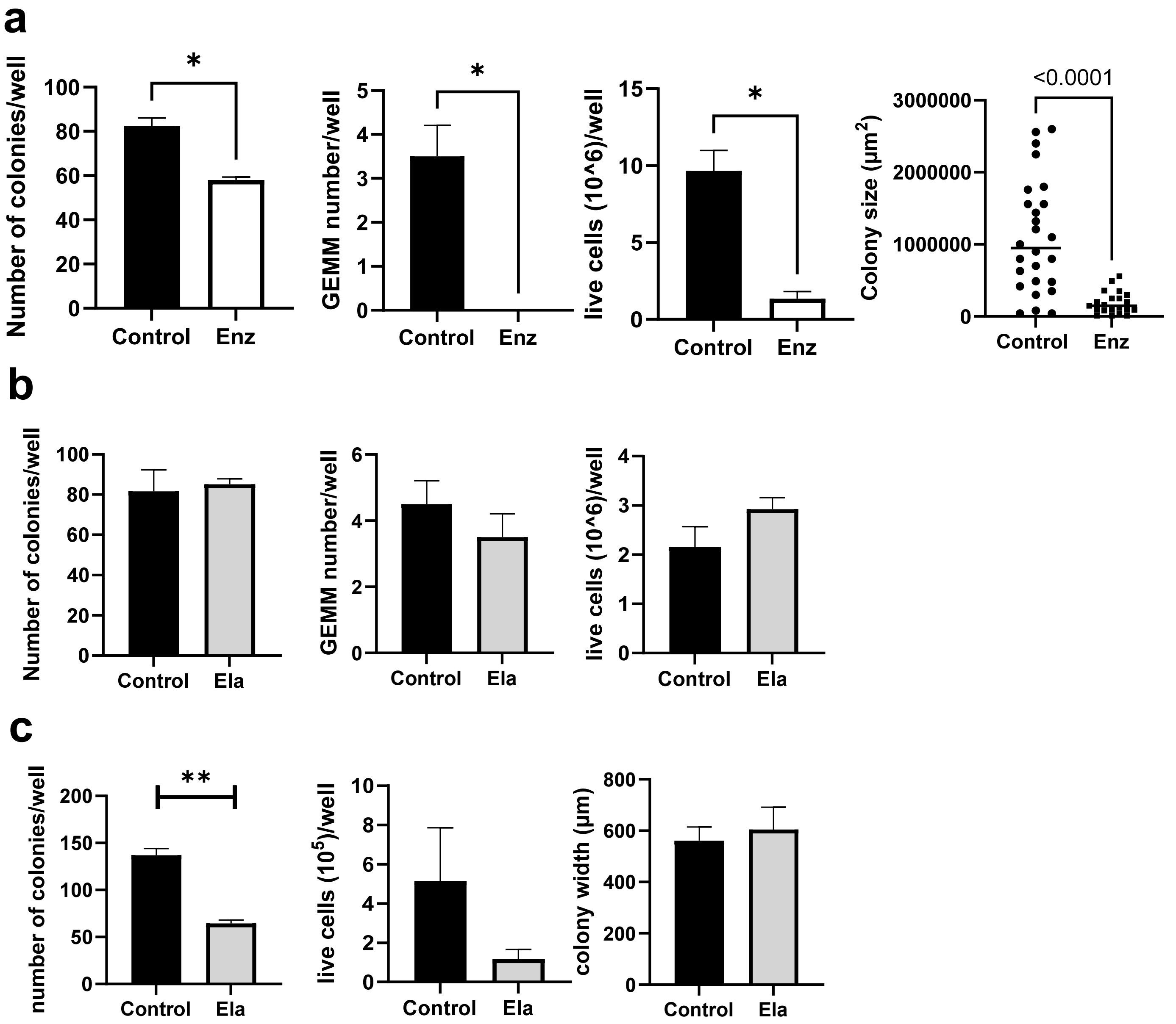

Supplementary Fig. 9 Effects of blocking sex hormone signaling on aging HSPCs.
(a) Effects of anti-androgen enzalutamide on methylcellulose colony formation of aging male BM cells. BM cells from 18-month-old male BALB/c mice were placed on methylcellulose medium with vehicle control or 50 µM enzalutamide (Enz) for the assay. Total colony number, GEMM (granulocyte, erythrocyte, megakaryocyte, monocyte) number, total live cell number and colony size were quantified. **(b, c)** Effects of anti-estrogen elacestrant on methylcellulose colony formation of BM cells from 18-month-old female BALB/c mice with treatment of 400 nM elacestrant (Ela) or vehicle control for the initial plating (b) and secondary plating (c). The concentrations of enzalutamide and elacestrant were physiological plasma concentrations seen in human patients. * P<0.05; ** P<0.01.

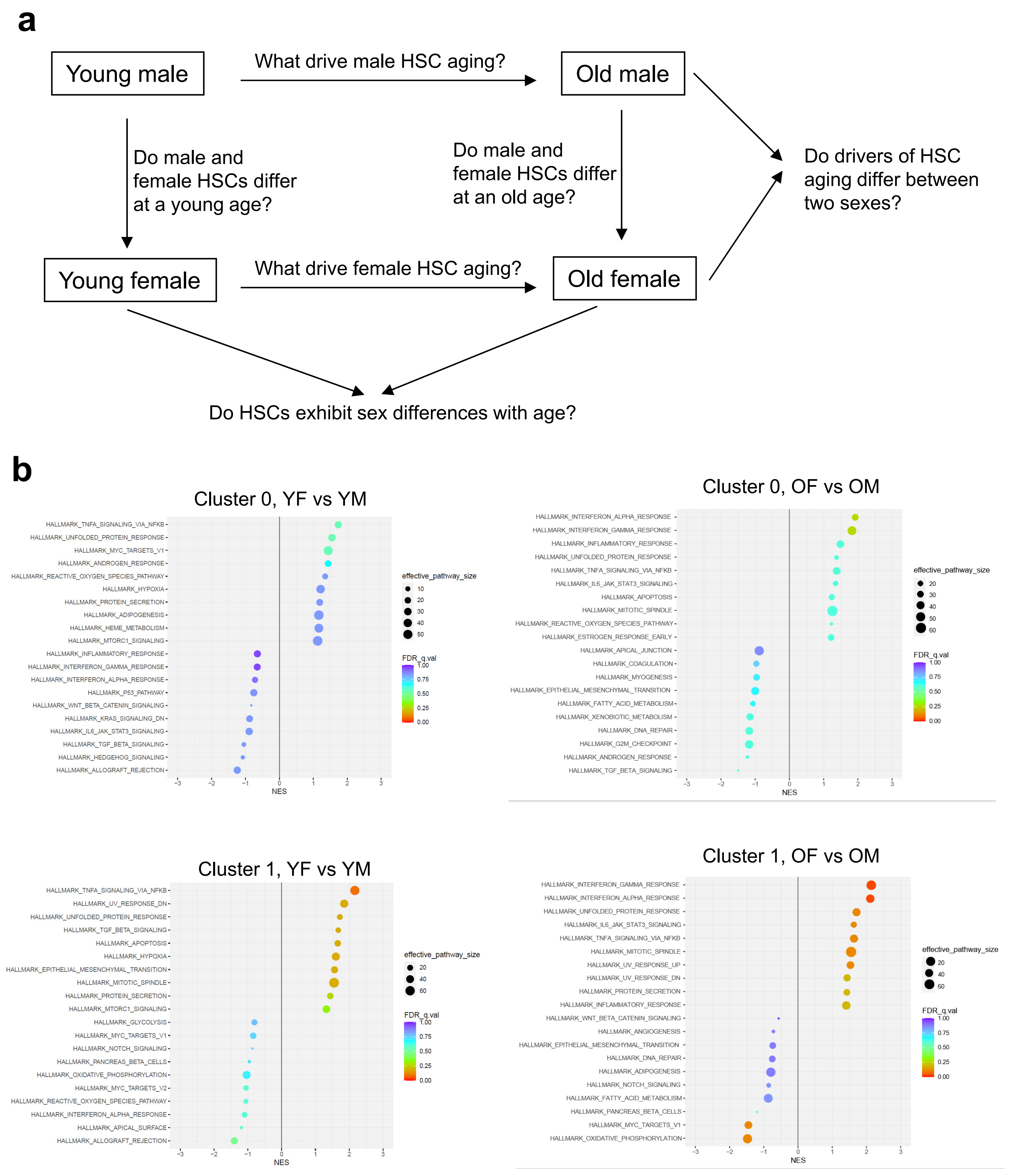

Supplementary Fig. 10

Supplementary Fig.10b (continued)

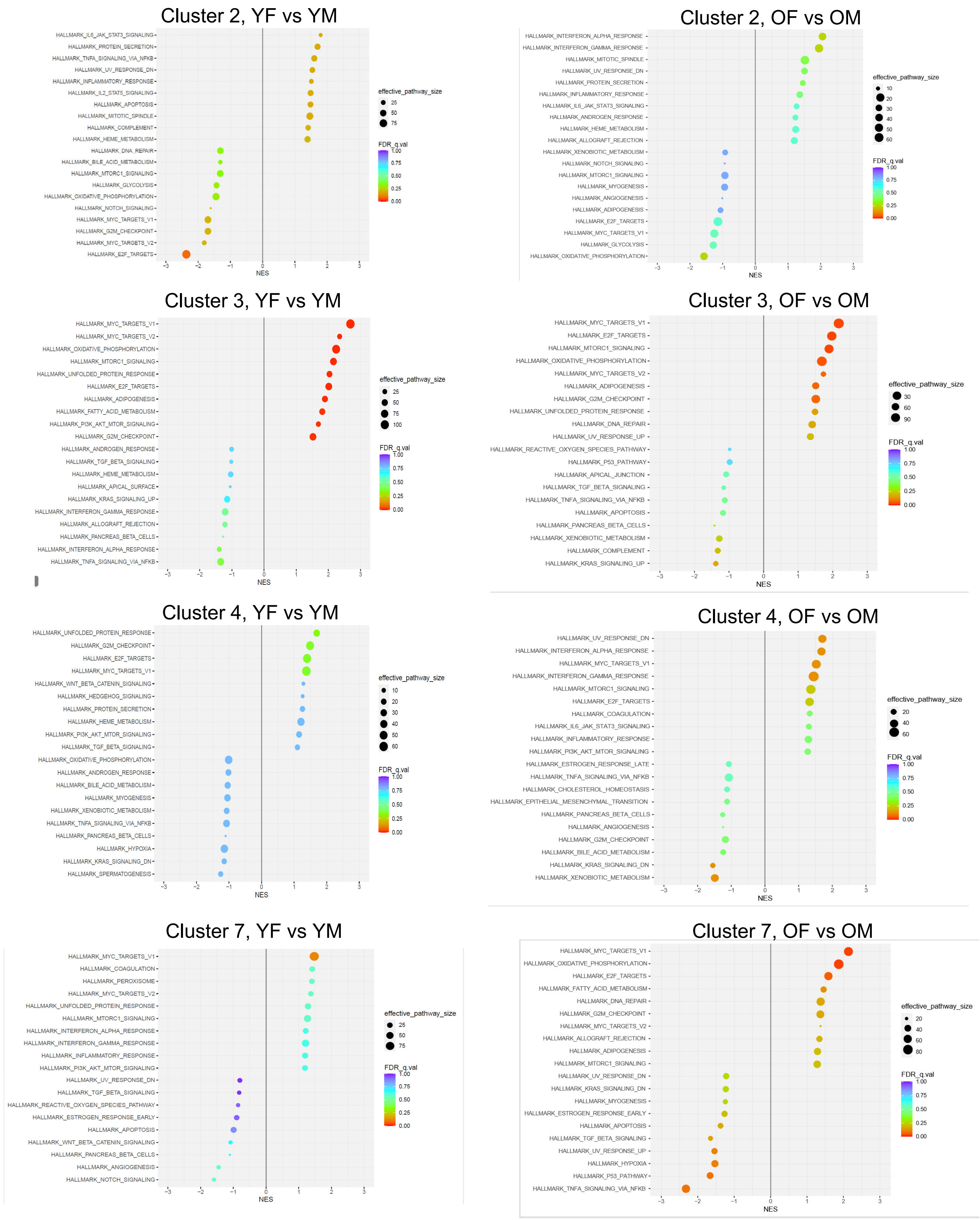

Supplementary Fig. 10 Analysis of sex and age differences of HSCs by different comparisons. **(a)** Scheme of HSC analyses for sex and age differences and related biological questions. **(b)** Analysis of female vs male LT-HSCs, ST-HSCs and progenitors at young and old ages. Cluster 5 was not available for comparison due to low cell counts in males.

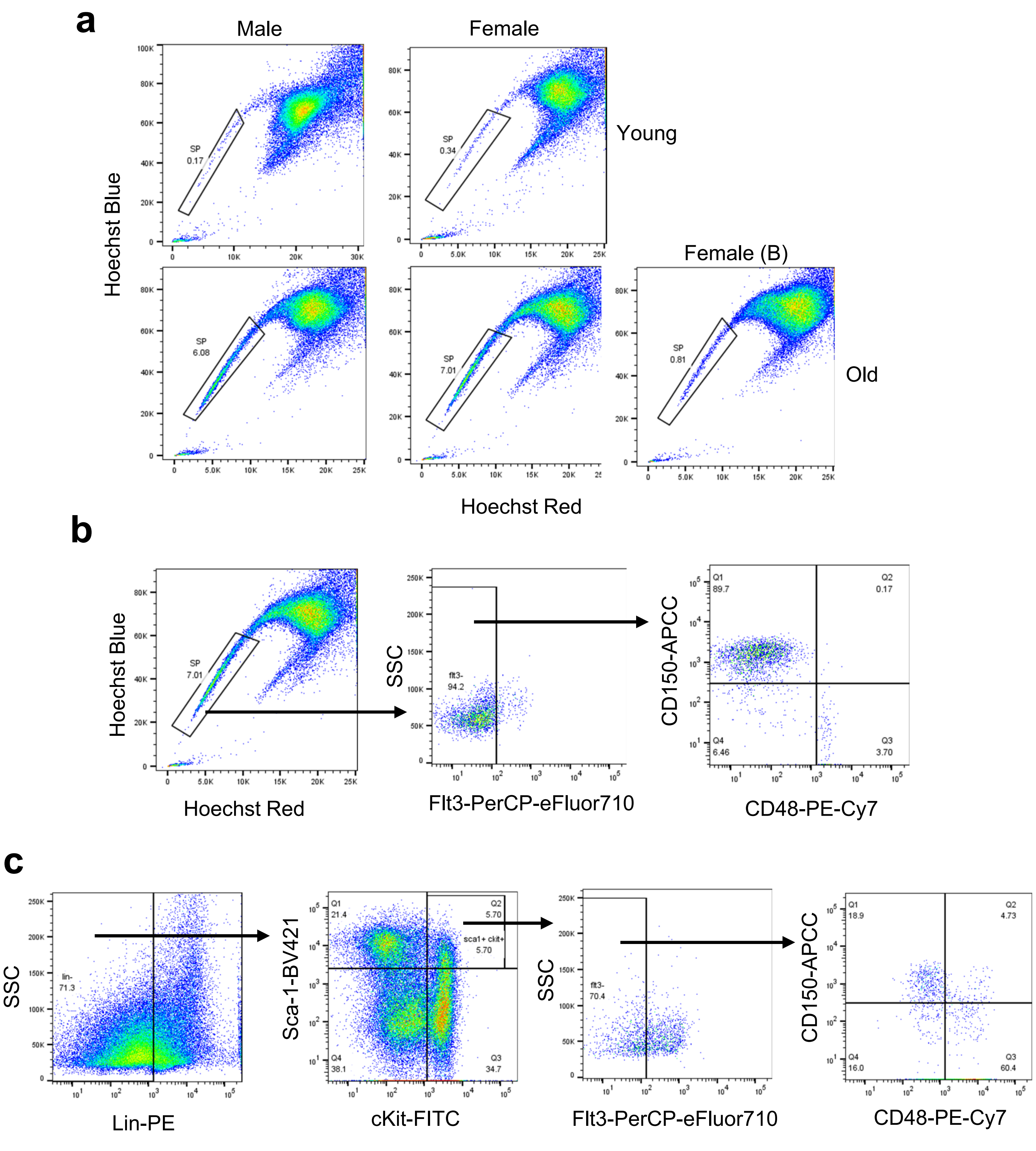

Supplementary Fig. 11 HSC analysis in C57BL/6 mice.
a Representative SP profiles of young (3 months) and old (27-28 months) C57BL/6 mice of two sexes. Old female (B) SP did not shift toward increasing lower SP. **b** Gating for LT-HSCs using SP-based method from lineage-depleted cells. **c** Gating for LT-HSCs using LSK-based method from lineage-depleted cells.

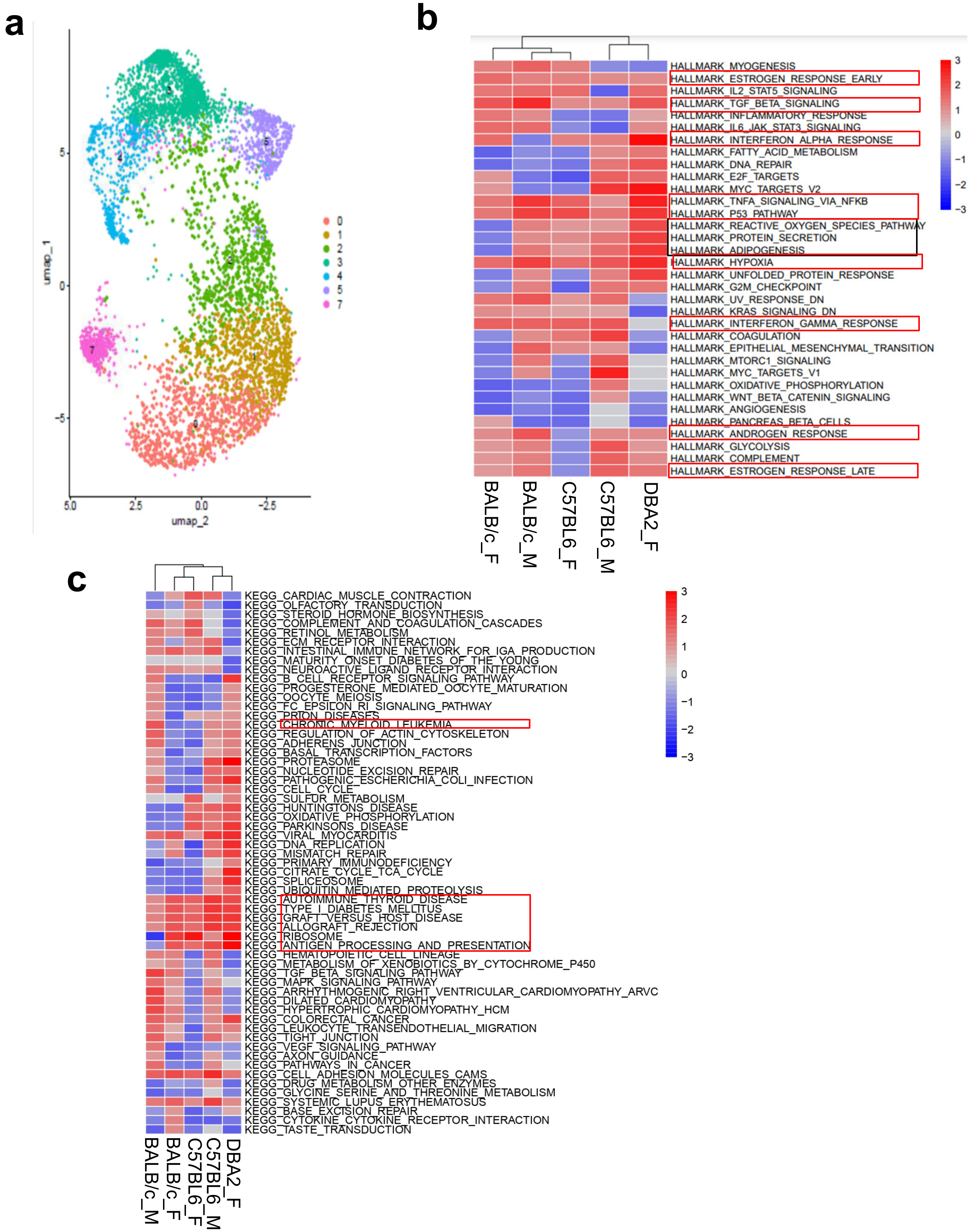

Supplementary Fig. 12 Comparison of HSC aging pathways in different mouse strains. **(a)** The GSE147729 dataset for C57BL/6 male scRNAseq data in the gene-barcode matrices in Market Exchange Format (MEX) were converted into Seurat objects, and then mapped onto BALB/c scRNAseq data of this study, which served as a reference. UMAP was created using the same parameters for BALB/c mice. **(b, c)** Cluster dendrograms for Hallmark (b) and KEGG (c) aging pathways of LT-HSCs from three mouse strains by combining datasets GSE59114 for C57BL/6 and DBA/2 females and GSE147729 for C57BL/6 males along with BALB/c data from the current study.
